# Antagonistic Effect of Sucrose Availability and Auxin on *Rosa* Axillary Bud Metabolism and Signaling, Based on the Transcriptomics and Metabolomics Analysis

**DOI:** 10.3389/fpls.2022.830840

**Published:** 2022-03-17

**Authors:** Ming Wang, Laurent Ogé, Maria-Dolores Pérez Garcia, Alexandra Launay-Avon, Gilles Clément, Jose Le Gourrierec, Latifa Hamama, Soulaiman Sakr

**Affiliations:** ^1^Dryland-Technology Key Laboratory of Shandong Province, College of Agronomy, Qingdao Agricultural University, Qingdao, China; ^2^Institut Agro, University of Angers INRAE, IRHS, SFR QUASAV, Angers, France; ^3^Institute of Plant Sciences Paris-Saclay (IPS2), CNRS, INRA, Université Paris-Sud, Université d’Evry, Université Paris-Saclay, Gif-sur-Yvette, France; ^4^Institut Jean-Pierre Bourgin, INRA, AgroParisTech, CNRS, Université Paris-Saclay, Versailles, France

**Keywords:** bud outgrowth, sucrose, auxin, signaling pathway, TOR-kinase

## Abstract

Shoot branching is crucial for successful plant development and plant response to environmental factors. Extensive investigations have revealed the involvement of an intricate regulatory network including hormones and sugars. Recent studies have demonstrated that two major systemic regulators—auxin and sugar—antagonistically regulate plant branching. However, little is known regarding the molecular mechanisms involved in this crosstalk. We carried out two complementary untargeted approaches—RNA-seq and metabolomics—on explant stem buds fed with different concentrations of auxin and sucrose resulting in dormant and non-dormant buds. Buds responded to the combined effect of auxin and sugar by massive reprogramming of the transcriptome and metabolome. The antagonistic effect of sucrose and auxin targeted several important physiological processes, including sink strength, the amino acid metabolism, the sulfate metabolism, ribosome biogenesis, the nucleic acid metabolism, and phytohormone signaling. Further experiments revealed a role of the TOR-kinase signaling pathway in bud outgrowth through at least downregulation of *Rosa hybrida BRANCHED1* (*RhBRC1*). These new findings represent a cornerstone to further investigate the diverse molecular mechanisms that drive the integration of endogenous factors during shoot branching.

## Introduction

Shoot branching is a particularly significant trait of survival and reproductive success for crops, but also of visual quality for ornamental plants (Garbez et al., 2015; Rameau et al., 2015; Barbier et al., 2019; Kotov et al., 2021). Lateral buds emerge at the leaf axils and remain dormant or become active to develop new branches. The resumption of bud growth is induced by removal of the fast-growing shoot apex involved in a mechanism called apical dominance (Dun et al., 2009; Müller and Leyser, 2011). Apical dominance currently appears as a network of hormone- and sugar-dependent mechanisms (Barbier et al., 2019; Schneider et al., 2019; Kotov et al., 2021). The hormonal regulatory network that controls shoot branching has been extensively studied (Rameau et al., 2015; Barbier et al., 2019; Maurya et al., 2020), while that of sugars is still nascent (Rabot et al., 2012; Barbier et al., 2015, 2021; Fichtner et al., 2017, 2020; Wang et al., 2019, 2021). In addition, the question on how hormonal and sugar pathways could interact to drive bud growth remains poorly investigated.

Auxin and sugars are two main systemic regulators with opposite roles in the control of bud outgrowth. Auxin is produced by young leaves at the shoot tip, transported basipetally *via* the polar auxin transport stream (PATS), and prevents axillary bud growth along the stem (Bennett et al., 2016; Harrison, 2017; Van Rongen et al., 2019). Auxin cannot enter the bud and acts through two non-mutually exclusive models referred to as the “second messenger” and “auxin canalization” models (Aguilar-Martínez et al., 2007; Prusinkiewicz et al., 2009; Müller and Leyser, 2011; Dun et al., 2012) supported by the two hormones cytokinins (CK) and strigolactones (SL; Brewer et al., 2013; Rameau et al., 2015; Barbier et al., 2019). Auxin impedes biosynthesis of CK, an inducer of axillary bud outgrowth operating partly through downregulation of *BRANCHED1* (*BRC1*), one of the major transcription factors involved in inhibition of branching (Braun et al., 2012; Dun et al., 2012). Conversely, auxin triggers synthesis of SL, a repressor of axillary bud outgrowth that partly acts *via BRC1* accumulation (Aguilar-Martínez et al., 2007; Braun et al., 2012; Dun et al., 2012). *BRC1* represses cell cycle activity and auxin export from bud to stem but stimulates biosynthesis of ABA, an inhibitor of shoot branching (González-Grandío and Cubas, 2014; González-Grandío et al., 2017; Shen et al., 2019).

As sink organs, buds have to compete with other plant sink organs to attract the sugar required for their high metabolic activity (Alves et al., 2007; Van den Ende, 2014). By diverting sugar routes, the fast-growing shoot apical meristem prevents axillary buds from importing sugar and thereby reduces their ability to grow out (Mason et al., 2014). Accordingly, dormant buds exhibit a carbon-starvation-like transcript profile (Tarancón et al., 2017). Conversely, bud outgrowth can be stimulated by increasing sugar supply to buds in whole plants or stem explants (Rabot et al., 2012; Barbier et al., 2015, 2021; Fichtner et al., 2017; Wang et al., 2021). A role of sugar in shoot branching has also been reported in other species (Liu et al., 2020).

Besides their trophic role, sugars are a signaling entity that promotes bud outgrowth (Rabot et al., 2012; Barbier et al., 2015, 2021; Fichtner et al., 2017, 2020; Wang et al., 2021), partially through repression of *BRC1* expression (Kebrom et al., 2010; Mason et al., 2014; Barbier et al., 2015; Kebrom and Mullet, 2015; Wang et al., 2019, 2021). Growing evidence indicates that sugar-dependent promotion of bud outgrowth involves several sugar-signaling-dependent pathways (Fichtner et al., 2017, 2020; Barbier et al., 2021), including glycolysis/the tricarboxylic acid (TCA) cycle and the oxidative pentose phosphate pathway (OPPP; Wang et al., 2021). Glycolysis/the TCA cycle provides cell energy (Kruger and Von Schaewen, 2003; Stincone et al., 2015; Christiaens et al., 2016; Cañas et al., 2017) and acts upstream of the target of rapamycin (TOR) kinase, a hub of sugar, and cell energy signaling (Brunkard et al., 2020; Burkart and Brandizzi, 2020). The contribution of TOR signaling in axillary bud outgrowth is still unknown, while it is required for shoot meristem activation (Xiong et al., 2013; Wu et al., 2019).

Sugar and auxin represent a highly complex and central signaling network that acts cooperatively in various aspects of plant development like cell proliferation and expansion, hypocotyl elongation, and root growth (Wang and Ruan, 2013; Min et al., 2014; Li et al., 2016; Sakr et al., 2018; Yuan et al., 2020). Conversely, sugar antagonizes auxin-dependent repression of bud outgrowth (Mason et al., 2014; Bertheloot et al., 2020) by impairing the SL-signaling pathway (Bertheloot et al., 2020; Patil et al., 2021). Glycolysis/the TCA cycle and the OPPP are an integrative part of this crosstalk (Wang et al., 2021). Based on these findings, we investigated whether the antagonistic effect of sugar and auxin on bud outgrowth could be extended to other components of bud metabolomics and signaling. Our results show that these two systemic regulators reshape bud metabolism and signaling, with a potential role of TOR kinase.

## Materials and Methods

### Plant Culture and *in vitro* Cultivation of Axillary Buds

*Rosa hybrida* L. cuttings were taken from cloned mother plants grown in the greenhouse as described by Barbier et al. (2015). At the visible floral bud (VFB) stage, axes plants were used to harvest stem node explants (single-node cuttings) from the median part of the stem and served for metabolomics and molecular analyses. Stem node segments (contain one bud) 1.5 cm in length were grown *in vitro* for 24 h on classical solid MS medium (Duchefa; 1% gelose, aubygel) supplemented with different concentrations of sucrose and 1-naphthaleneacetic acid (NAA; a synthetic auxin), resulting in three different states of dormancy (Bertheloot et al., 2020): dormant buds (Suc10 + NAA; 10 mM sucrose +1 μM NAA), partially dormant buds (Suc10; 10 mM sucrose and Suc100 + NAA; 100 mM sucrose +1 μM NAA), and non-dormant buds (Suc100; 100 mM sucrose).

Single-node cuttings incubated on sugar-containing medium (S10 or S100) were also fed with different concentrations of AZD8055 (1 and 10 μM), a main potent effector of TOR-kinase activity (Montané and Menand, 2013; Inaba and Nagy, 2018). These buds were used to investigate the involvement of TOR kinase in bud outgrowth by analyzing their ability to grow out and characterizing the expression level of BRC1.

### RNA Extraction and RT–qPCR

Total RNA was extracted from stem node segments (0.5 cm) or stably transformed *Rosa* calluses (40 mg) using an RNA NucleoSpin kit (Macherey-Nagel) according to the manufacturer’s recommendations, with slight modifications (Barbier et al., 2015). The absence of contamination by genomic DNA was monitored by PCR using a specific primer designed against an intron region of the *RhGAPDH* gene (Girault et al., 2010; Henry et al., 2011). cDNAs were obtained by reverse transcription performed on 1 μg of RNA using SuperScript III Reverse Transcriptase (Invitrogen, Inc.). Quantitative real-time RT-PCR (RT-qPCR) was performed with SYBR Green Supermix (Bio-Rad, Inc.) using cDNA as a template, following the protocol from Barbier et al. (2015). Relative expression of different genes (Supplementary Table S1) was quantified using *RhUBC* and *RhSAND* as internal controls (Jain et al., 2006; Chua et al., 2011). Specific sets of primers were selected according to their melting curves (Supplementary Table S2).

### RNA-seq Library Construction and Sequencing

Three independent biological replicates were produced for each treatment (Suc10, Suc10 + NAA, Suc100, and Suc100 + NAA). RNA samples were obtained from 50 stem node explant buds incubated for 24 h. RNA-seq libraries were built following the manufacturer’s recommendations (TruSeq-Stranded-mRNA-SamplePrep-Guide-15031047D-protocol, Illumina®). The RNA-seq samples were sequenced in paired-end (PE) with a read length of 75 bases. The raw data (fastq) were trimmed with the Trimmomatic tool (Bolger and Giorgi, 2014), and ribosome sequences were removed with the sortMeRNA tool (Kopylova et al., 2012). The STAR genomic mapper (Dobin et al., 2013) was used to align reads against the *Rosa chinensis* genome (Saint-Oyant et al., 2018). Dispersion was estimated using edgeR (Robinson et al., 2010). Expression differences were compared using the likelihood ratio test, and *p*-values were adjusted using the Benjamini-Hochberg procedure to control the false discovery rate (FDR). Fragments per kilobase of transcripts per million fragments mapped (FPKMs) were calculated for visual analysis only and were obtained by dividing normalized counts by gene length.

### Metabolomics Analysis

Three independent biological replicates were produced for each treatment (Suc10, Suc10 + NAA, Suc100, and Suc100 + NAA). For each treatment, 50 buds from stem node segments were collected at 48 h incubation. The frozen buds taken from one-node cuttings were re-suspended in 1 ml of frozen (−20°C) water:acetonitrile:isopropanol (v/v/v, 2:3:3) containing Ribitol at 4 μg·ml^−1^ and extracted for 10 min at 4°C with shaking at 1,400 rpm in an Eppendorf Thermomixer. Insoluble material was removed by centrifugation at 20,000 *g* for 5 min. Fifty microliters were collected and dried overnight at 35°C in a speed-vac and stored at −80°C. The same steps were followed for three blank tubes as a negative control. Metabolomics experiments were performed as in Clément et al. (2017), in which all steps were performed according to Fiehn (2006) and Fiehn et al. (2008).

### Promoter Cloning, Stable Rose Callus Transformation, and GFP Fluorescence Analysis

The *RhBRC1* promoter (1,973 bp upstream of the start codon) was cloned, and rose calluses were stably transformed as described by Wang et al. (2021). Three different assays of stably transformed calluses were incubated on a range of AZD8055 concentrations (0.5, 1, 5, and 10 μM) for 8 h. The transcription levels of GFP in the calluses treated or not with different concentrations of AZD8055 have been monitored as described by Wang et al. (2021).

### Measurements of Vacuolar Invertase Activity

Enzyme activity was evaluated as described by Girault et al. (2010) and Rabot et al. (2012). For each sample, an extract was obtained by grinding frozen tissues (100 mg of bud) in the extraction buffer [50 mM HEPES-NaOH (pH 7.0), 10 mM MgCl_2_, 1 mM Na_2_EDTA, 2.6 mM DTT, 10% ethylene glycol (v/v), and 0.02% Triton X-100 (v/v)], giving a final volume of 2 ml. The extract was centrifuged for 3 min in a microcentrifuge (12,000 *g*, 4°C) and the supernatant was desalted on a G25 Sephadex column (GE Healthcare). Enzyme activity was assayed on 25 μl of desalted extract supplemented with 2 μl of 0.2 M sodium acetate (pH 4.8). Ten microliter of 0.6 M sucrose were added, and the reaction was allowed to proceed at 30°C for 20 min. Then, the reaction was stopped by adding 50 μl of 0.5 M NaH_2_PO_4_ (pH 7.0). The samples were incubated for 3 min at 100°C and placed on ice. Then, they were mixed with 750 μl of a reaction buffer composed of 50 mM HEPES-NaOH (pH 7.0), 2 mM MgCl_2_, 1 mM EDTA, 100 mM sucrose, 1 mM ATP, 0.4 mM NAD, 4.2 U of hexokinase, 3.5 U of phosphoglucoisomerase, and 2 U of glucose-6-*P*-dehydrogenase, and were incubated at 30°C for 20 min. NADH formation was monitored spectrophotometrically at 340 nm. The amount of proteins in each extract was measured by the Bradford method (1976) with bovine serum albumin as a standard (Bradford, 1976).

### Statistical Analyses

R software was used for statistical treatment. One-way ANOVA (*α* = 0.05) was run to test for the effects of the different conditions on bud outgrowth, gene transcription, and fluorescence. The samples followed a normal distribution. Significant differences were indicated by different letters or asterisks directly on the figures.

## Results

### Transcriptomic Profiling of Axillary Buds Exposed to the Combined Effect of Auxin and Sugar

**Figure 1:**
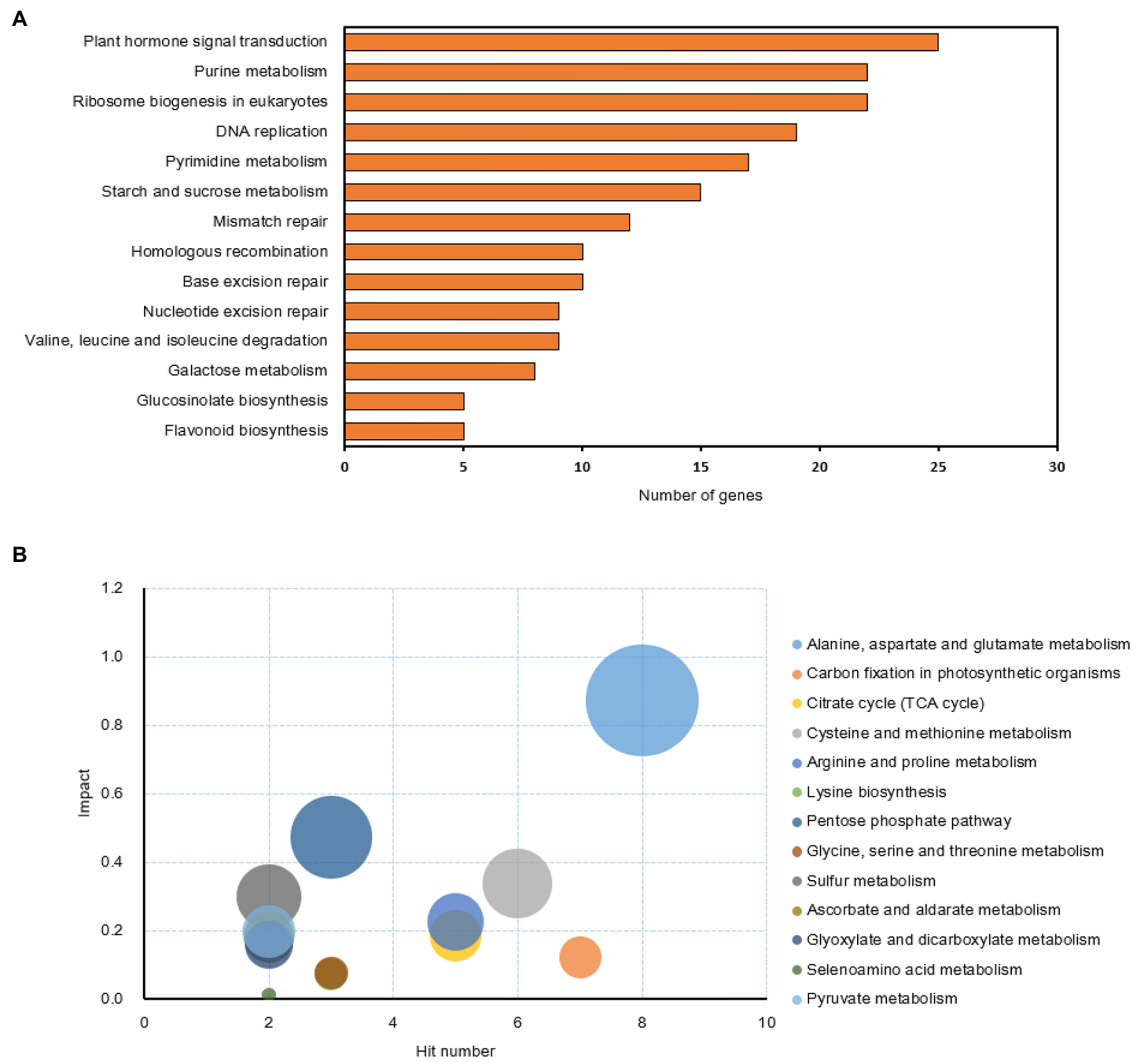
KEGG analysis of the genes and compounds involved in the antagonistic effect of sucrose and auxin in buds treated for 24 h. **(A)** KEGG pathway analysis of the antagonistic-related genes (*p* < 0.05) based on ClusterProfiler analysis (Yu et al., 2012); **(B)** KEGG pathway analysis of the compounds (*p* < 0.05) influenced by the antagonistic effect of sucrose and auxin based on MetaboAnalyst database analysis (Chong and Xia, 2018).

Previous experiments showed that sucrose can relieve the inhibiting effect of synthetic auxin (NAA, 1-naphthaleneacetic acid) during bud outgrowth of *Rosa hybrida* (Bertheloot et al., 2020; Wang et al., 2021). To get a comprehensive insight into the early mechanisms driving this antagonistic crosstalk, RNA-seq and metabolomics experiments were conducted on axillary buds from one-node stem explants treated with four conditions of sucrose and auxin (Suc10, Suc10 + NAA, Suc100, and Suc100 + NAA) and harvested before the onset of outgrowth (Wang et al., 2021). Based on RNA-seq data from 24 h-treated buds, differentially expressed genes (DEGs) ranged from 2,430 to 5,568 in pairwise comparisons of the different conditions (Supplementary Figure S1). The relationships between the different DEG groups were displayed as Venn diagrams, and the transcription of 1,418 genes (654 downregulated by auxin and upregulated by sucrose, 764 upregulated by auxin and downregulated by sucrose) were under the antagonistic control of sucrose and auxin (Supplementary Figure S2). In addition, a metabolomics analysis conducted on 48 h-treated buds (no change was found earlier at *t* = 24 h) produced 106 known metabolites in axillary buds, including amino acids, intermediates of the sugar metabolism, organic acids, and secondary metabolites (Supplementary Figure S3). Only 46 metabolites were under the antagonistic effect of auxin and sucrose; among them, 40 were stimulated by sucrose, while six were stimulated by auxin. KEGG analysis was performed using the ClusterProfiler package in R software and the MetaboAnalyst database (Yu et al., 2012; Chong and Xia, 2018) to determine the function of these DEGs. We found 25 and 22 genes involved in plant hormone signal transduction and ribosome biogenesis, respectively. Nineteen genes were involved in DNA replication, and 22 and 17 participated in the purine and pyrimidine metabolisms, respectively (Figure 1A). Besides the starch and sugar metabolisms (Figures 1A,B; Wang et al., 2021), the amino acid (alanine, aspartate and glutamate, cysteine and methionine, lysine biosynthesis, glycine, serine, and threonine) metabolism was also under the antagonistic effect of sucrose and auxin (Figure 1B). Interestingly, the sulfur metabolism was also affected, supporting a tight link between the sugar, amino acid, and sulfur metabolisms (Schmidt and Jäger, 1992). As for secondary metabolism, only the metabolism of glucosinolates and flavonoids responded to the combined effect of auxin and sugar. These findings indicate that the combined effect of auxin and sugar results in a significant reprograming of the transcriptome and metabolome of axillary buds in Rosa.

### Response of Bud Sink Strength Markers to the Combined Effect of Auxin and Sugar Availability

**Figure 2:**
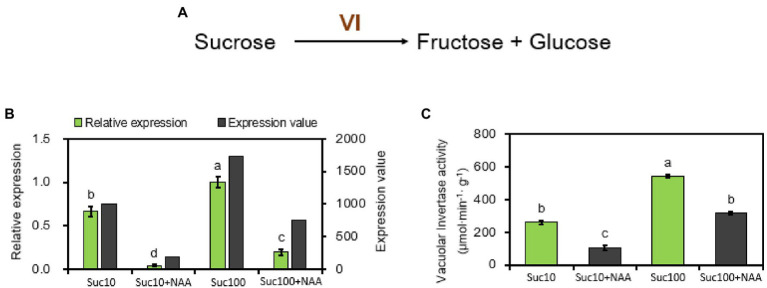
Vacuolar invertase (*RhVI1*) is regulated by auxin (NAA) and sucrose (Suc) in stem explant buds. **(A)** Catalysis pathway mediated by RhVI1; **(B)** Expression level of *RhVI1* (dark and light green columns correspond to RNA-seq and RT-qPCR results respectively), RNA-seq, and RT-qPCR results with sucrose alone (Suc10, Suc 100) and with auxin and sucrose (Suc10 + NAA and Suc100 + NAA); and **(C)** total activity of *RhVI1* in buds supplied with sucrose only (Suc10 and Suc100) or NAA combined with sucrose (Suc10 + NAA and Suc100 + NAA). Data are the means ± SEs of three biological replicates. Suc10, 10 mM sucrose; S10N, 10 mM sucrose + 1 μM NAA; S100, 100 mM sucrose; and S100N, 100 mM sucrose + 1 μM NAA. Letters, significant differences between the different treatments with *p* < 0.05.

**Figure 3:**
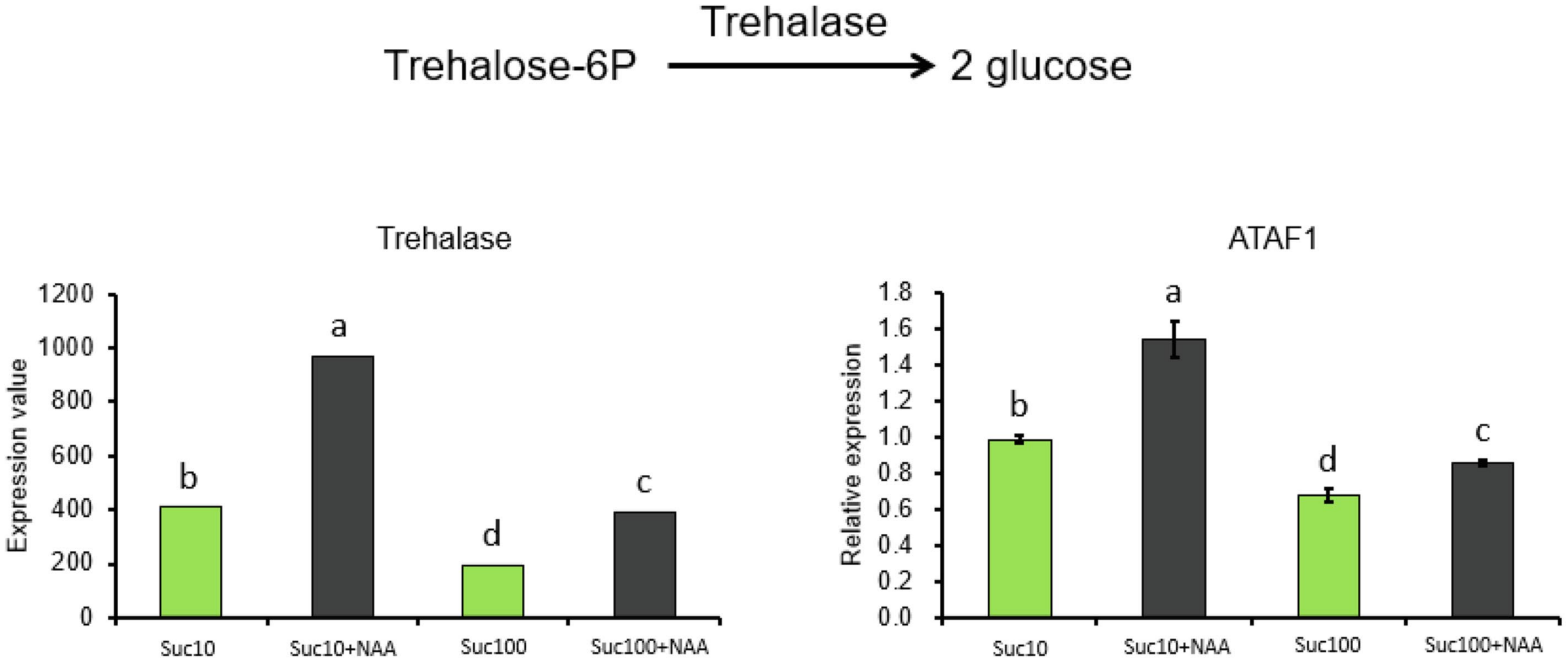
Expression level of No APICAL MERISTEM/ARABIDOPSIS TRANSCRIPTION ACTIVATOR FACTOR/CUP-SHAPED COTYLEDON (*ATAF1*) and *Trehalase* in *in vitro*-cultured buds supplied with different treatments. Suc10, 10 mM sucrose; Suc10 + NAA, 10 mM sucrose + 1 μM NAA; Suc100, 100 mM sucrose; and Suc100 + NAA, 100 mM sucrose + 1 μM NAA. Data are the means ± SEs of three biological replicates. Letters, significant differences between the different treatments with *p <* 0.05.

Vacuolar invertase (VI) is an important enzyme of the sucrose metabolism and in the establishment of the strength of diverse sink organs (Nägele et al., 2010; Morey et al., 2018). It catalyzes sucrose cleavage to produce glucose (Glc) and fructose (Fru) in the vacuole (Figure 2A). *Rosa hybrida* vacuolar invertase 1 (*RhVI1*) plays a pivotal role in bud outgrowth promotion because it is tightly correlated with bud ability to grow out (Girault et al., 2010; Rabot et al., 2012). Our RNA-seq and RT-qPCR results showed that its expression level and activity were oppositely regulated by auxin (NAA) and sucrose (Figure 2B). *RhVI1* expression was greatly stimulated by rising sucrose concentrations (1.7-fold higher in 100 mM than in 10 mM sucrose-fed buds) but highly repressed by NAA (Figure 2B). Auxin-dependent *RhVI1* repression was 4.7 times stronger than in dormant (Suc10) and partially dormant (Suc100 + NAA) buds and was correlated with its enzymatic activity (Figure 2C). Therefore, *RhVI1* could be a key player of bud sink strength targeted by the combined effect of auxin and sucrose. In addition, two markers of the depletion of cellular carbon and energy pools in plants—the transcription level of *ATAF1* and trehalase—emerged from RNA-seq to be responsive to the combined effect of auxin and sugar availability. *ATAF1* is an *Arabidopsis thaliana* No APICAL MERISTEM/ARABIDOPSIS TRANSCRIPTION ACTIVATOR FACTOR/CUP-SHAPED COTYLEDON (NAC) transcription factor that directly and transcriptionally upregulates the expression of trehalase, which breaks trehalose down into two molecules of glucose (Garapati et al., 2015). The expression of these two genes was correlated with the dormancy status of the buds; their highest level was exhibited by dormant buds (Suc10 + NAA) while their lowest level was found in non-dormant ones (Suc100; Figure 3). Partially dormant buds (Suc10 and Suc100 + NAA) exhibited an intermediary level. Taken together, these data indicate that auxin and sugar act antagonistically on the sink strength of buds to drive their ability to grow out.

### Response of the Amino Acid Metabolism to the Combined Effect of Auxin and Sugar

**Figure 4:**
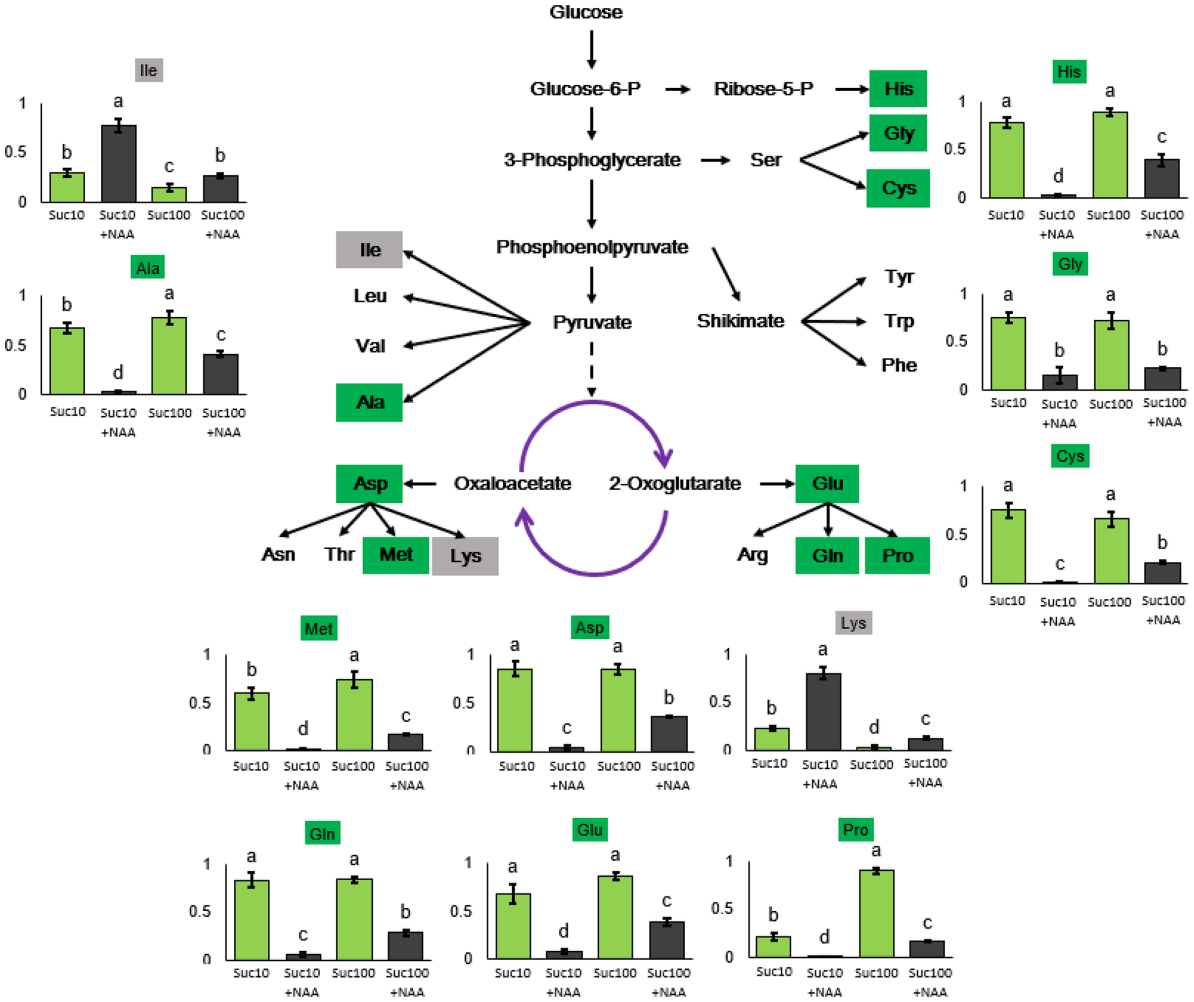
Amino acid levels in axillary buds following different treatments. Suc10, 10 mM sucrose; Suc10 + NAA, 10 mM sucrose +1 μM NAA; Suc100, 100 mM sucrose; and Suc100 + NAA, 100 mM sucrose +1 μM NAA. Dark green label, amino acid inhibited by auxin but stimulated by sucrose [nine amino acids: Ala (alanine), Asp (aspartate), Met (methionine), Glu (glutamate), Gln (glutamine), Pro (proline), His (histidine), Gly (glycine), and Cys (cysteine)]. Gray label, amino acid inhibited by sucrose, but stimulated by auxin [Ile (isoleucine) and Lys (lysine)]. Green and dark columns, sucrose- and sucrose+auxin-treated buds, respectively. Data are means ± standard errors (SEs). Letters, significant differences between the different treatments with *p <* 0.05.

Our previous results demonstrated that glycolysis/the TCA cycle—the main carbon supplier of the amino acid metabolism—was regulated by the antagonistic effect of sucrose and auxin (Wang et al., 2021). To determine whether amino acid synthesis was also impaired, RNA sequencing data and amino acids from the metabolomics analysis were selected and analyzed (Figure 4). RNA-seq results indicated that auxin tended to promote the expression of 17 genes coding for amino-acid-degrading enzymes, while sucrose positively affected the expression of eight genes encoding amino-acid-metabolism-related enzymes (Supplementary Figure S4). In addition, the content in nine amino acids (alanine, histidine, aspartate, methionine, proline, glutamate, glutamine, cysteine, and glycine) was significantly reduced by auxin (Suc10 + NAA) and partially alleviated by high sucrose availability (Suc100 + NAA). The reverse action (upregulated by auxin and downregulated by sucrose) was only limited to two amino acids—lysine and isoleucine (Figure 4). The lysine content in arabidopsis seeds was found inversely correlated with TCA-cycle activity, and an enhanced lysine content caused delayed germination (Angelovici et al., 2011). Proline was the sole amino acid whose content was both greatly responsive to sugar availability (4.1 times higher in buds fed with 100 mM sucrose than in those fed with 10 mM sucrose) and to the combined effect of auxin and sucrose (Figure 4). Proline is biosynthetically derived from the amino acid L-glutamate and is dependent on cellular reducing power (NADH and NADPH) generated by the OPPP (Kruger and von Schaewen, 2003), which is under the control of the combined effect of auxin and sugar in buds (Wang et al., 2021).

### Response of the Sulfate Metabolism to the Combined Effect of Auxin and Sugar

**Figure 5:**
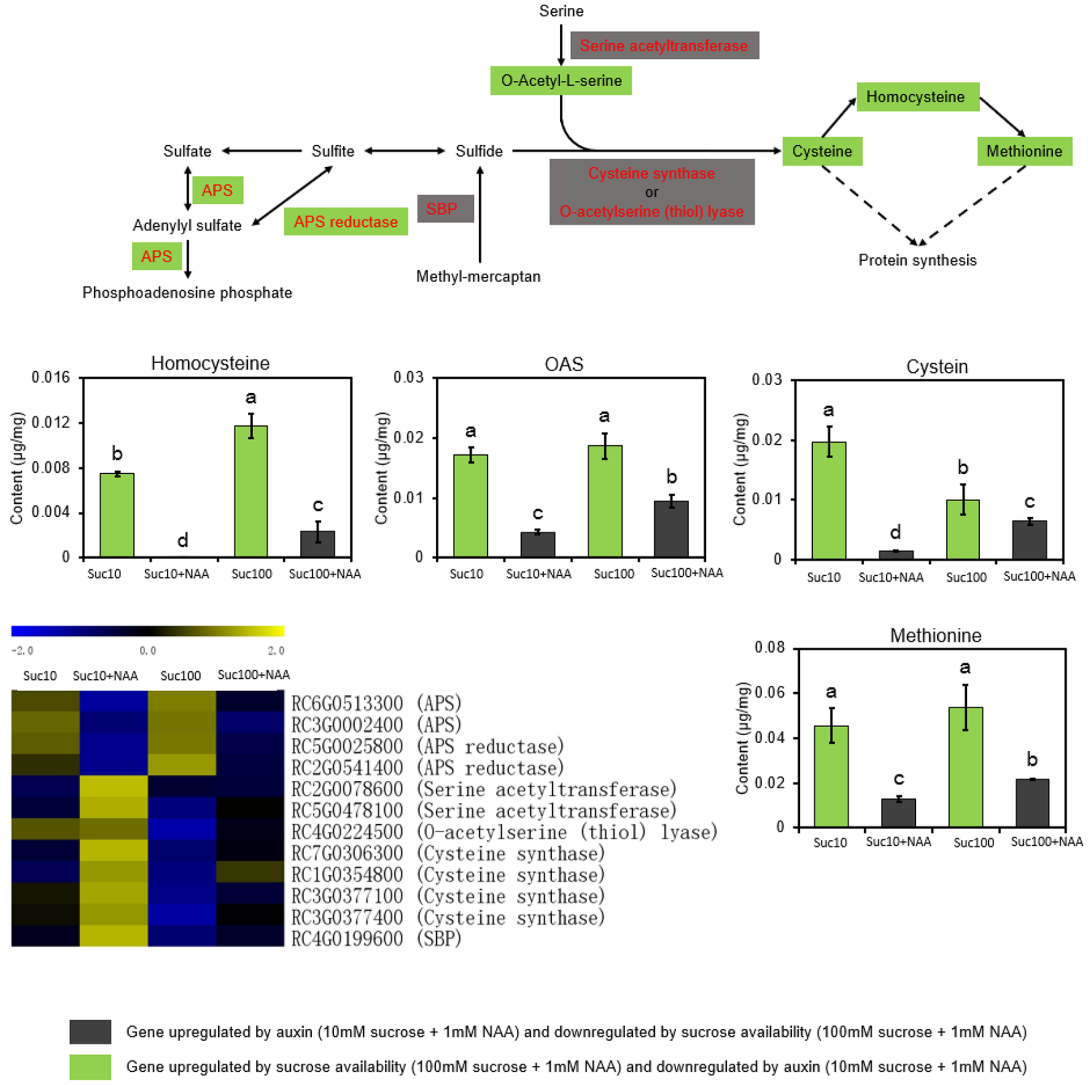
The sulfur metabolism is antagonistically regulated by auxin and sugar in axillary buds. Suc10, 10 mM sucrose; Suc10 + NAA, 10 mM sucrose + 1 μM NAA; Suc100, 100 mM sucrose; and Suc100 + NAA, 100 mM sucrose + 1 μM NAA. Dark green labels, enzymes-encoding genes (APS (ATP-sulfurylase) and APR [5′-adenylylsulfate (APS) reductase)] or metabolites (O-acetylserine, cysteine, and homocysteine) repressed by auxin but stimulated by sucrose. Gray labels, enzymes-encoding genes (serine acetyltransferase; cysteine synthase; and selenium-binding protein, SBP) repressed by sucrose but stimulated by auxin. Data are the means ± standard errors (SEs). Letters, significant differences between the different treatments with *p* < 0.05. Green bars, effect of sucrose alone; gray bars, combined effect of auxin and sucrose. Heatmap color (from blue to yellow), gene transcription level from low to high, respectively.

Sulfur (S) is an essential macronutrient for plant growth, development, and response to environmental changes. As the first committed step of sulfate assimilation, ATP-sulfurylase (ATP-S or APS) catalyzes sulfate activation and yields activated high-energy adenosine-5′-phosphosulfate that is reduced to sulfide by APS reductase (5′-Adenylylsulfate (APS) reductase; APR), before being incorporated into cysteine (Anjum et al., 2015; Figure 5). Cysteine is the precursor of a huge number of sulfur-containing metabolites essential for the metabolism and the antioxidant function of plants (Takahashi et al., 2011). The sulfur metabolism was significantly impaired by auxin, and this effect was partially alleviated by sucrose (Figure 5). Dormant buds (Suc10 + NAA) exhibited downregulated transcript levels of APS and APR, along with a low content of several sulfur-containing compounds (cysteine, homoserine, methionine, and O-acetylserine (OAS)) compared with partially (Suc10; Suc100 + NAA) and non-dormant buds (Suc100; Figure 5). In addition, only the expression level of APS and APR was positively associated with sugar availability. This was in line with earlier feeding experiments showing that sugar stimulates the transcript level, protein level, and activity of APR in arabidopsis roots (Hesse et al., 2003). On the contrary, partially and completely dormant buds displayed downregulated expression of serine acetyltransferase and cysteine synthase (O-acetylserine (thiol) lyase), which are involved in the final step of cysteine biosynthesis (Noji and Saito, 2002). These two enzymes do not appear to catalyze the rate-limiting steps of sulfate assimilation during bud outgrowth because their repression does not impede the ability of buds to grow out. In sum, the cellular metabolism of buds, including the sugar, amino acid, and sulfur metabolisms, is impaired by the inhibitory effect of auxin, and this effect is mitigated by sucrose availability.

### Response of Ribosome Biogenesis to the Combined Effect of Auxin and Sugar

**Figure 6:**
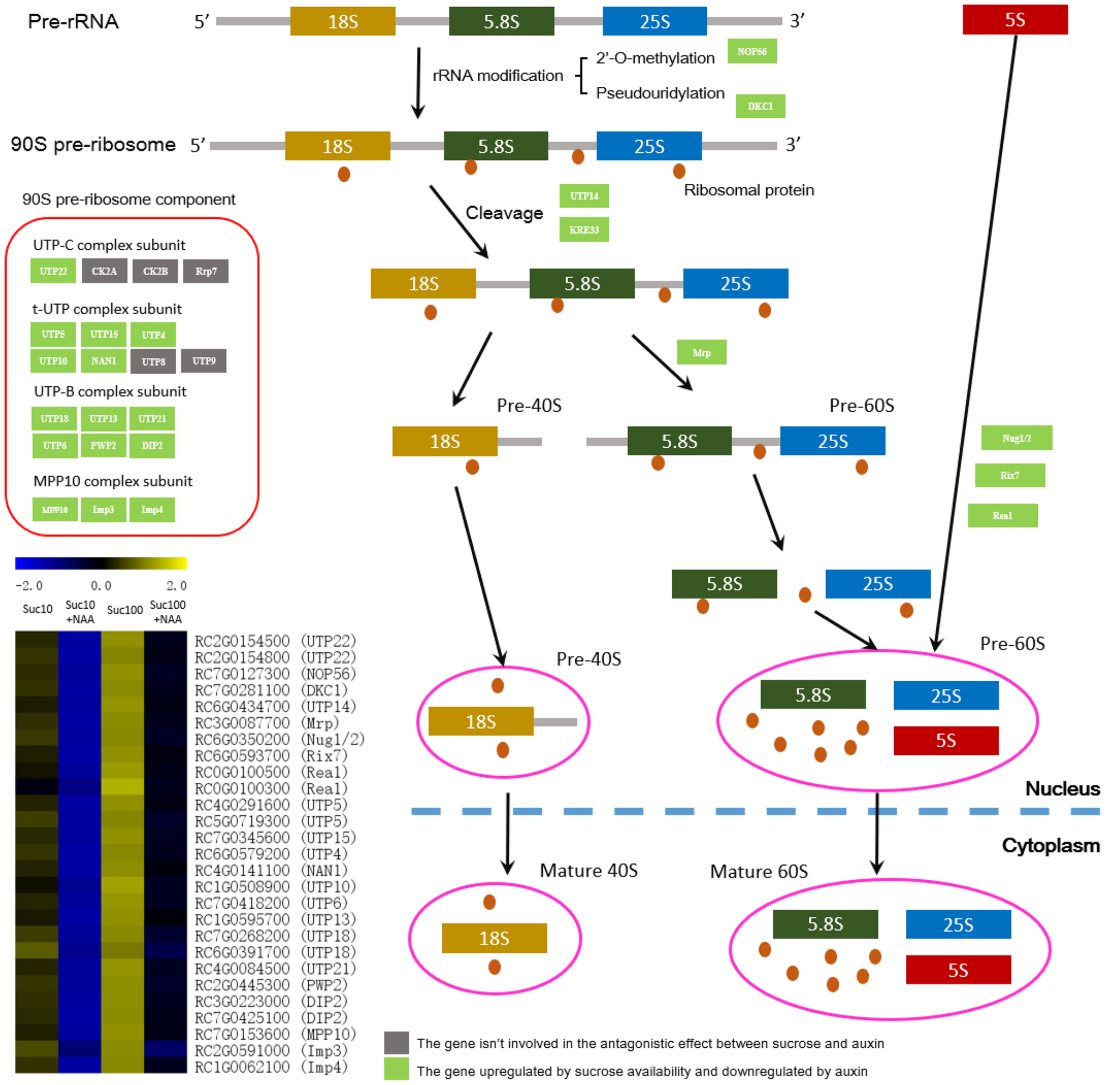
The ribosome biosynthesis machinery is affected by the antagonistic effect of sucrose and auxin. Suc10, 10 mM sucrose; Suc10 + NAA, 10 mM sucrose + 1 μM NAA; Suc100, 100 mM sucrose; and Suc100 + NAA, 100 mM sucrose + 1 μM NAA. Dark green labels, enzymes-encoding genes repressed by auxin but stimulated by sucrose. Gray labels, enzymes-encoding genes repressed by sucrose but stimulated by auxin. Heatmap color (from blue to yellow), gene transcription level from low to high, respectively.

Ribosome biogenesis, an energy-consuming process, is a key process of protein synthesis and is closely linked to the main cellular activities including cell proliferation, differentiation, and growth (Kressler et al., 2010; Weis et al., 2015; Denise et al., 2019). Being a high-energy-consuming process (Warner, 1999; Du and Stillman, 2002), ribosome biogenesis might be downregulated in dormant buds. In line with this, the abundance of genes related to RNA biogenesis was repressed by auxin in dormant buds, and this effect was alleviated by sugar availability (Figure 6). Several genes including 90S pre-ribosome components—such as the t-UTP complex subunit, the UTP-B complex subunit, and the MPP10 complex subunit (Kornprobst et al., 2016)—and those involved in ribosomal RNA (rRNA) modification—*NOP56* and *DKC1* (Tran et al., 2003; Mochizuki and Gorovsky, 2004)—and cleavage—*UTP14* and *KRE33* (Dragon et al., 2002)—were downregulated by auxin in dormant buds, and this effect was partially attenuated by high sugar availability (Figure 6). These findings are in agreement with the low energy status of dormant buds compared to active ones (Wang et al., 2021). In addition, other key genes of ribosome biogenesis including *ribonuclease P/MRP protein subunit* (*Mrp*), *nuclear GTP-binding protein* (*Nug1/2*), *ribosome biogenesis ATPase* (*Rix7*), and *midasin-like protein* (*Real*) were antagonistically regulated by auxin and sucrose, and downregulated by auxin (Figure 6).

### Response of the Nucleic Acid Metabolism to the Combined Effect of Auxin and Sugar

**Figure 7:**
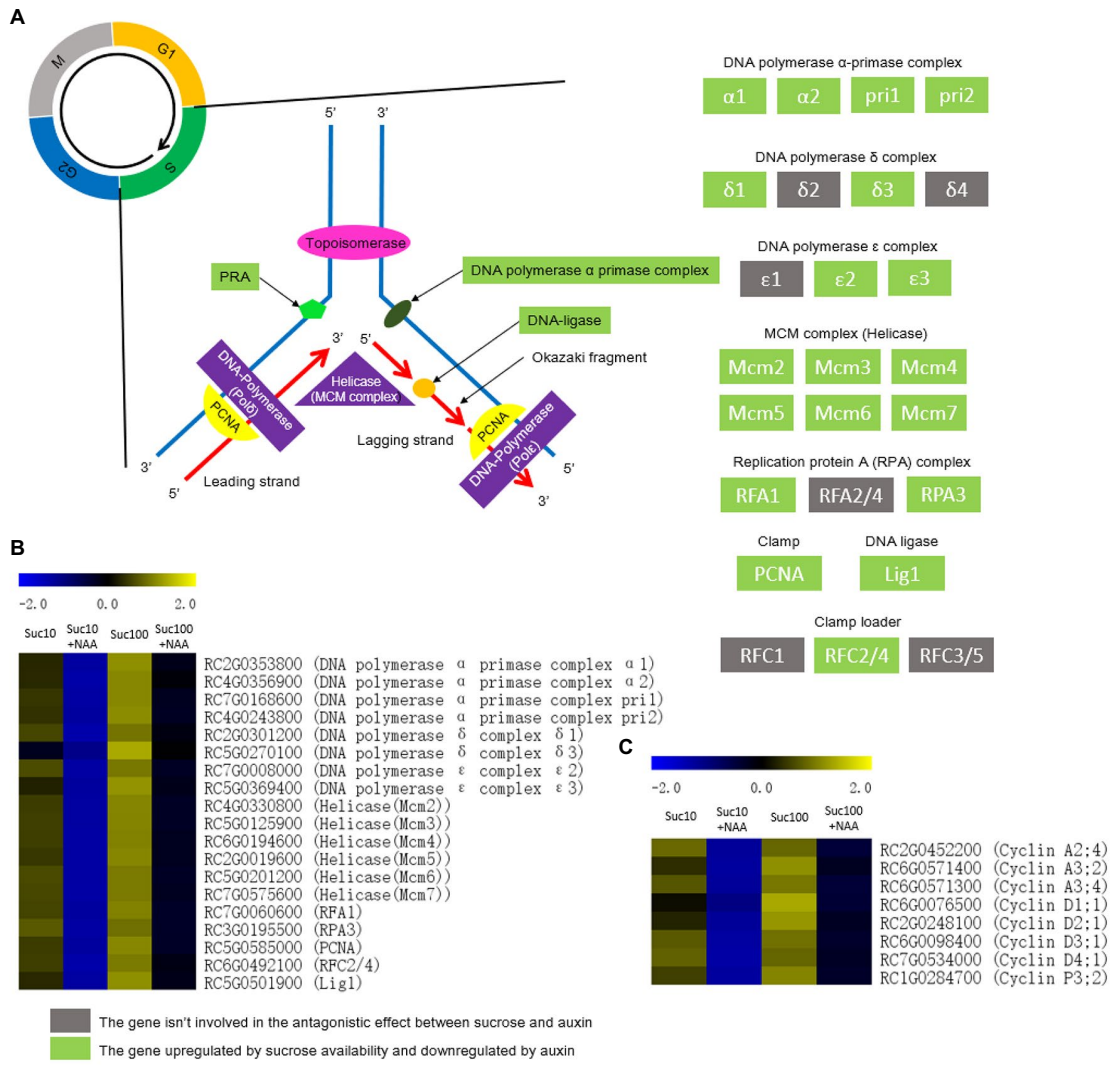
DNA replication and cell division are antagonistically regulated by auxin and sugar in axillary buds. Suc10, 10 mM sucrose; Suc10 + NAA, 10 mM sucrose +1 μM NAA; Suc100, 100 mM sucrose; and Suc100 + NAA, 100 mM sucrose +1 μM NAA. **(A)** Sucrose and auxin antagonistically regulate the transcription level of many key factors of DNA replication; **(B)** several genes that encode the key factors of DNA replication are regulated by the antagonistic effect of sucrose and auxin; and **(C)** several genes related to the cell cycle are regulated by the antagonistic effect of sucrose and auxin. Dark green labels, enzymes-encoding genes repressed by auxin but stimulated by sucrose. Gray labels, enzymes-encoding genes repressed by sucrose but stimulated by auxin. Heatmap color (blue to yellow), gene transcription level (low to high, respectively).

DNA replication is highly energy-consuming and strongly responsive to the plant nutrient status (Shultz et al., 2007; Polyn et al., 2015). The transcription level of the components of the DNA polymerase complex (Pols) and of the maintenance of mini chromosomes (MCM) complex was under the regulation of the antagonistic effect of auxin and sugar availability (Figures 7A,B). Pols are divided into at least six different complexes named alpha, beta, gamma, delta, epsilon, and zeta that are central players in DNA replication, DNA damage repair, control of cell cycle progression, chromatin remodeling, and epigenetic regulation (Franklin et al., 2001; Pedroza-García et al., 2017). The antagonistic effect of auxin and sucrose significantly concerned most of the components of the DNA polymerase α-primase complex, the DNA polymerase δ complex, and the DNA polymerase ε complex (Figures 7A,B). MCM proteins serve as a licensing factor for DNA replication during phase S of the cell mitotic cycle (Tuteja et al., 2011). They form heterohexameric complexes (MCM2-7) that are all downregulated in dormant buds (Figures 7A,B). Similarly, dormant buds exhibit downregulated replication of the protein A (RPA) complex, and of an AAA+ ATPases required for DNA replication, repair, and recombination (Aklilu and Culligan, 2016; Figures 7A,B). Nucleotide biosynthesis is deeply reliant on ribose 5-phosphate, an important precursor of pyrimidine ribonucleotide synthesis (Zrenner et al., 2006). The ribose 5-phosphate content was lower in dormant buds than in active ones (Supplementary Figure S5; Wang et al., 2021).

The transcription of eight genes encoding cyclins (mainly cyclins A2, A3, and D1, 2, 3, and 4) was also controlled by the antagonistic effect of sucrose and auxin (Figure 7C). These genes are an integrative part of the cell cycle machinery. They are repressed by auxin in dormant buds, while their transcript level is recovered in response to high sucrose availability. In arabidopsis, sugar deprivation of meristem-induced cell cycle arrest (Francis and Halford, 2006) and two cyclins D (CycD2 and CycD3) acts as direct mediators of the presence of sugar in cell cycle commitment (Riou-Khamlichi et al., 2000). These findings indicate that the antagonistic effect of auxin and sugar is complex and takes place at different stages of the cell proliferation process.

### Response of Hormone Synthesis and Signaling to the Combined Effect of Auxin and Sugar

We identified three main plant hormones related to the regulatory network of bud outgrowth: the auxin biosynthesis and signaling pathway, and the abscisic acid (ABA) and cytokinin signaling pathways.

**Figure 8:**
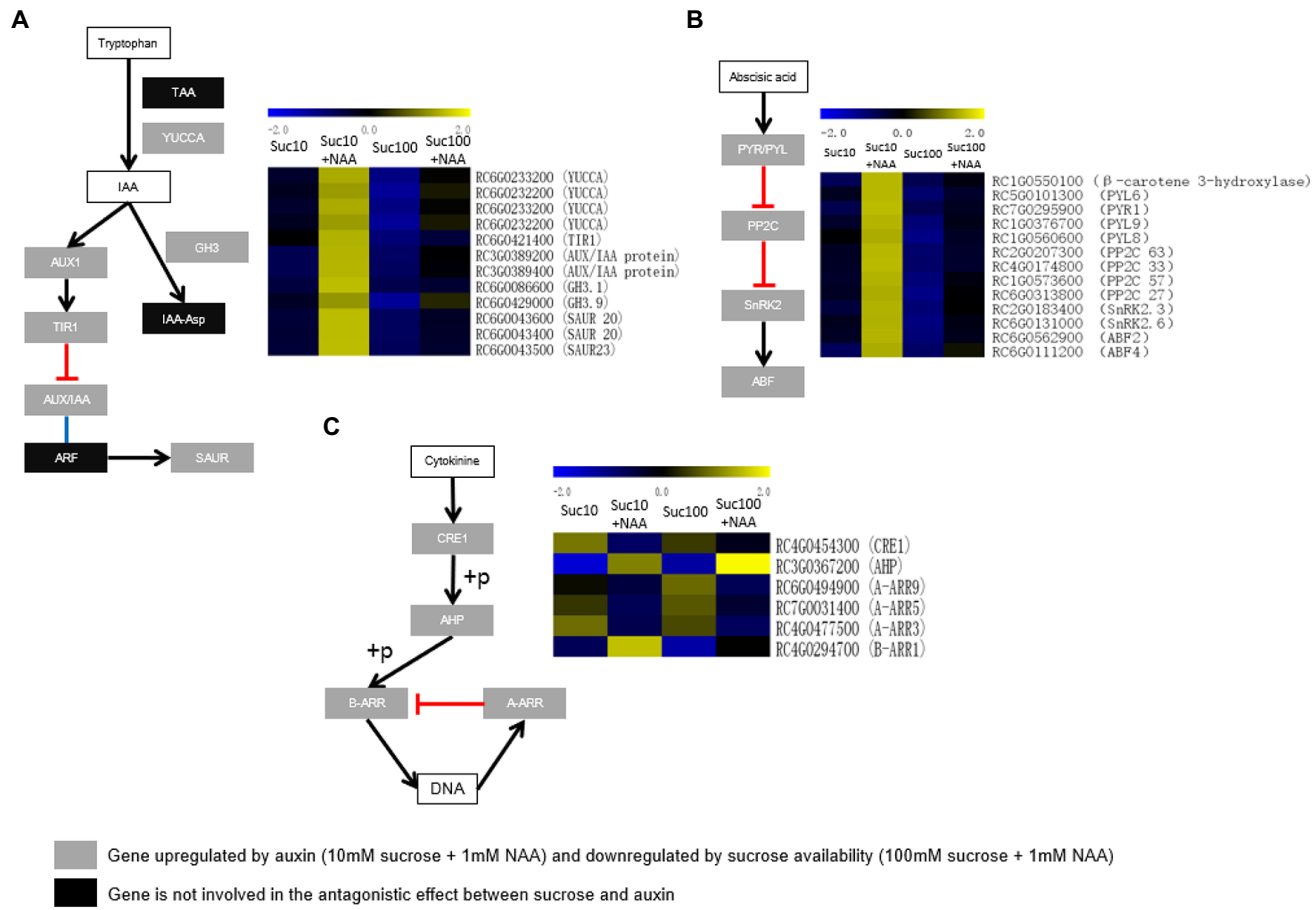
Expression patterns of **(A)** the genes related to auxin biosynthesis (*TAA* and *YUCCA*), conjugation to amino acid (*GH3*), transport (*AUX1*), and signaling (*TIR1*, *AUX/IAA*, *ARF*, and *SAUR*); **(B)** the genes related to abscisic acid signaling (*PYR/PYL*, *PP2C*, *SnRK2*, and *ABF*), and **(C)** the genes related to cytokinin signaling (*CRE1*, *AHP*, *A-RR*, and *B-RR*) in response the combined effect of auxin and sucrose in buds. S10, 10 mM sucrose; S10N, 10 mM sucrose + 1 μM NAA; S100, 100 mM sucrose; and S100N, 100 mM sucrose + 1 μM NAA. Dark green labels, enzymes-encoding genes repressed by auxin but stimulated by sucrose. Gray labels, enzymes-encoding genes repressed by sucrose but stimulated by auxin. Heatmap color (blue to yellow), gene transcription level (low to high, respectively).

Auxin (IAA) is the major negative systemic regulator of bud outgrowth (Barbier et al., 2019). Its level is lower in dormant buds than in non-dormant buds of *Rosa* sp. (Barbier et al., 2015). In plants, IAA is mainly synthesized by two-step pathway. TRYPTOPHAN AMINOTRANSFERASE OF ARABIDOPSIS (TAA) family enzymes convert tryptophan (Trp) into indole-3-pyruvic acid (IPA), and flavin monooxygenase (YUCCA) family enzymes catalyze the subsequent transformation of IPA into IAA (Aoi et al., 2020). The transcription level of four genes encoding YUCCA was stimulated by auxin but inhibited by sucrose (Figure 8A). IAA is metabolized into corresponding amino acid conjugates by auxin-amido synthetases (auxin-conjugating enzymes) encoded by the GRETCHEN HAGEN 3 (GH3) genes (Park et al., 2007). Two genes encoding GH3 were upregulated in dormant buds (Figure 8A). The metabolomics analysis showed that the tryptophan content was lower in buds fed with sucrose but remained uninfluenced by auxin supply (Supplementary Figure S3). Dormant buds exhibited upregulated auxin transport and signaling, in line with the observed lower auxin content. The transcription level of *AUX1*, encoding a carrier protein involved in proton-driven auxin influx (Swarup and Bhosale, 2019), and of three main components of the auxin signaling pathway—TRANSPORT INHIBITOR RESPONSE 1/AUXIN SIGNALING F-BOX (TIR1) and two AUXIN/IAA (auxin/indole-3-acetic acid) proteins—was higher in dormant buds (Figure 8A). Three genes encoding the small auxin up RNA (SAUR; Figure 8A), corresponding to the largest family of early auxin-responsive genes in higher plants, are also upregulated in dormant buds (Zhang et al., 2021). These findings indicate that auxin homeostasis and signaling within buds are under the combined effect of auxin and sugar availability.

Abscisic acid (ABA) is involved in many plant developmental processes, including as a repressor of bud outgrowth (Barbier et al., 2019). The ABA signaling network is likely more sensitive than the ABA biosynthesis network to the antagonistic effect of auxin and sucrose availability. Only β-carotene hydroxylase, an enzyme of the zeaxanthin biosynthesis pathway required for ABA biosynthesis (Hao et al., 2010), was consistently stimulated in dormant buds (Figure 8B). By contrast, all the constituents of the ABA signaling network were upregulated in dormant buds and downregulated in partially active and fully active buds, including ABA receptors [PYR(pyrabactin resistance)/PYL(PYR1-like)], type-2C protein phosphatases (PP2Cs), SNF1-related protein kinase 2 (SnRK2), and ABA-responsive elements binding factor (ABF; Figure 8B). Upon ABA perception, pyrabactin resistance (PYR)/PYR1-like (PYL) inhibits the activity of PP2Cs (Santiago et al., 2012), resulting in the auto-phosphorylation of SnRK2 required for ABF activation (Fujita et al., 2013). This regulation involves four genes encoding PYR/PYL, four genes encoding the PP2C protein family, two genes encoding the SnRK2 family, and two genes encoding ABF2 and ABF4, two key components of ABA signaling (Rushton et al., 2012). These findings indicate that the ABA signaling network could be at the core of the antagonistic effect of auxin and sugar on bud outgrowth.

Cytokinins (CK) are promoters of bud outgrowth (Rameau et al., 2015; Barbier et al., 2019; Wang et al., 2021) and act as repressors of *BRC1* expression (Dun et al., 2012). The transcription level of many key factors of the CK signaling pathway, such as cytokinin receptor 1 (*CRE1*), histidine phosphotransfer proteins (*AHP*), 3 type-A response regulators (*A-RR3, 5* and *9*), and one type-B response regulator (*B-RR1*), were antagonistically regulated by auxin and sucrose (Figure 8C). More interestingly, type-B RR1, whose ortholog in arabidopsis is linked to bud outgrowth repression (Waldie and Leyser, 2018), was upregulated by auxin but repressed by sucrose availability. By contrast to auxin, sucrose promoted the accumulation of three type-A-RRs (RR3, 5 and 9; Figure 8C), in accordance with the reduced branching phenotype of the sextuple mutant lacking the clade of type-A RRs in arabidopsis (*arr3,4,5,6,7,15*; Müller et al., 2015). These results support that hormone signaling may be more sensitive to the antagonistic effect of auxin and sugar, in agreement with our previous data on the SL pathway (Bertheloot et al., 2020).

### Response of Bud Outgrowth and *BRC1* Expression to TOR-Kinase Inhibitors

**Figure 9:**
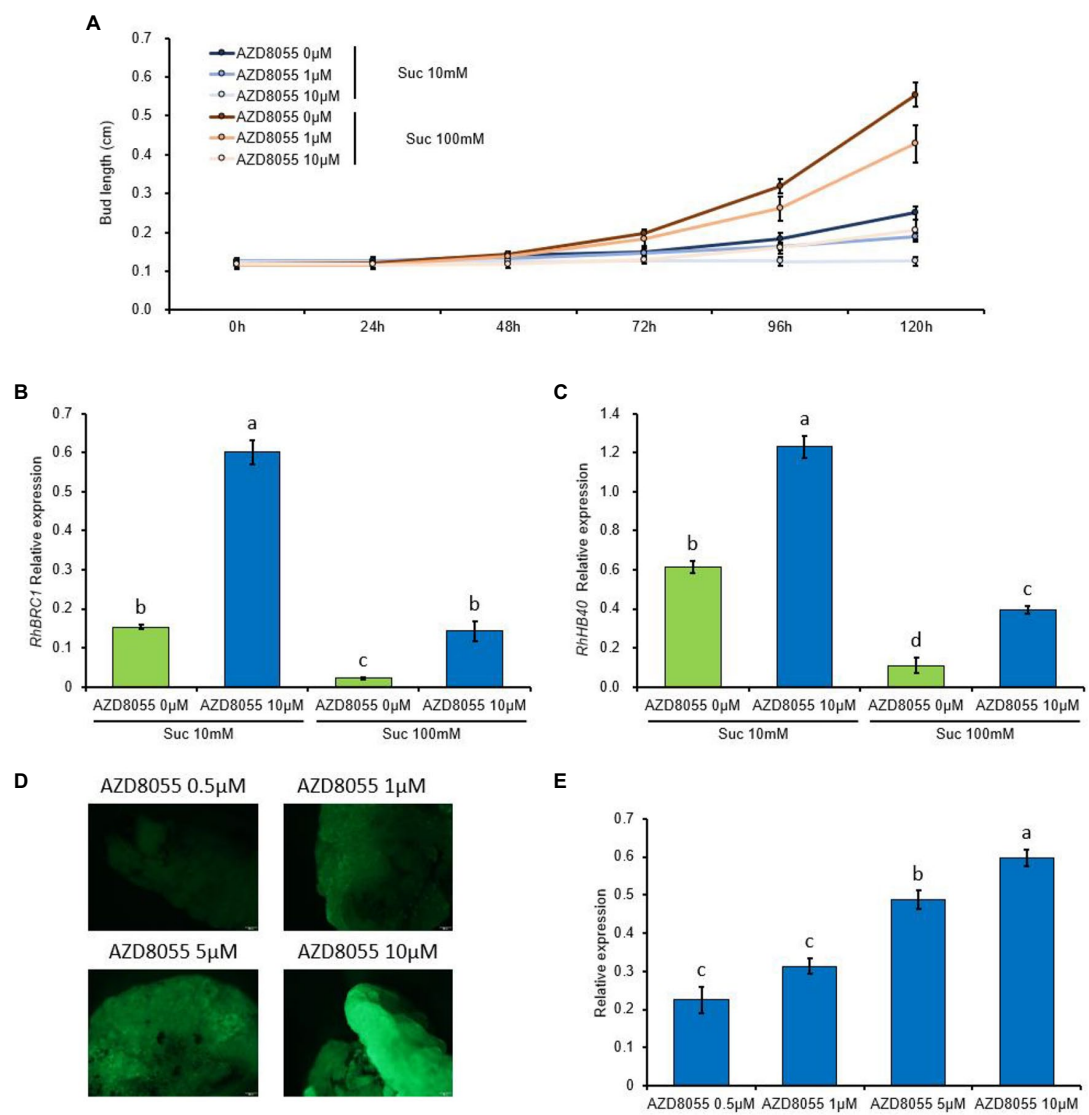
TOR kinase regulates bud outgrowth and *RhBRC1* expression through its promoter region. **(A)** Lengths of buds treated with different sucrose concentrations and AZD8055 (50 buds *per* treatment); **(B,C)** transcription levels of *RhBRC1* and *RhHB40*, respectively; **(D)** fluorescence levels of the 1973 bp-*RhBRC1* promoter in stably transformed calluses placed on different concentrations of AZD8055; and **(E)** transcription levels of *GFP* in the calluses treated with different concentrations of AZD8055. Green bars, sucrose treatment alone; blue bars, combined supplementation of sucrose and AZD8055. Data are the means of three biological replicates ± standard error (SE). Letters, significant differences between the different treatments with *p <* 0.05.

TARGET OF RAPAMYCIN (TOR) kinase signaling is at the core of the crosstalk between nutrient availability/the energy status and plant growth/development (Brunkard et al., 2020; McCready et al., 2020). Our previous data clearly demonstrated that auxin and sugar availability regulated bud outgrowth by acting antagonistically on glycolysis/the TCA cycle—the main energy provider for buds—while dormant buds exhibited sugar starvation (Wang et al., 2021). These findings and the fact that several TOR-kinase-inducible processes (the nucleic acid metabolism, protein translation, and ribosome biogenesis) are all downregulated in dormant buds prompted us to investigate whether TOR signaling could be required for bud outgrowth. First, the ability of buds to grow out was monitored by using stem node segments grown *in vitro* on classical solid medium (Rabot et al., 2012; Barbier et al., 2015; Wang et al., 2021) supplemented or not (control) with a concentration range of TOR-kinase inhibitor (AZD8055). AZD8055 feeding of the buds reduced their ability to grow out in a concentration-dependent manner (Figure 9A): bud length decreased as the AZD8055 concentration increased. High sucrose availability (100 mM) partially relieved this effect (Figure 9A); therefore, the effect depended on sugar availability. Elongation was completely inhibited when the buds were fed with 10 μM AZD8055 and 10 mM sucrose.

We also tested the transcription level of *RhBRC1* (a master repressor of bud outgrowth) and *RhHB40* (an HD-Zip transcription factor transcriptionally and directly controlled by AtBRC1, Whipple et al., 2011; González-Grandío et al., 2017; Dong et al., 2019), used as a marker of the transcriptional activity of *RhBRC1*. In accordance with our previous data (Wang et al., 2021), the transcript levels of *RhBRC1* and *RhHB40* were both reduced in response to sugar availability (Figures 9B,C). The addition of AZD8055 increased the transcription level of *RhBRC1* and *RhBH40*, much more so when the buds were incubated on a low sucrose concentration (10 mM; Figures 9B,C). Taken together, these findings support that *RhBRC1* accumulation in buds is regulated by AZD8055, and this regulation is negatively associated with TOR-kinase activity.

We recently showed that the promoter of *RhBRC1* was a converging site of sugar signaling that regulated *BRC1* expression transcriptionally (Wang et al., 2021). To check whether the promoter of *RhBRC1* played a central role in TOR-kinase-mediated downregulation of *RhBRC1*, the full-length promoter of *RhBRC1* (1,973 bp, pRhBRC1) was isolated and fused to the GFP-coding sequence in expression vectors stably transformed into *Rosa* calluses (Wang et al., 2021). After 8 h of incubation of AZD8055-fed medium, the GFP fluorescence and GFP transcript levels of the calluses stably transformed with the −1,973 bp promoter (pRhBRC1::GFP) increased in a concentration-dependent manner (Figures 9D,E). The highest level of GFP transcript was found with 10 μM AZD8055, indicating that the *BRC1* promoter could be part of TOR-kinase-dependent signaling.

## Discussion

Auxin and sugar are one of the main systemic regulators of shoot branching that act antagonistically to regulate bud outgrowth (Mason et al., 2014; Barbier et al., 2015; Bertheloot et al., 2020; Wang et al., 2021). However, the regulatory mechanism networks involved in this opposite crosstalk are still elusive because auxin and sucrose are generally reported to drive a variety of physiological plant processes cooperatively (Hartig and Beck, 2006; Mishra et al., 2021). According to the trophic hypothesis of apical dominance, auxin leads to photoassimilate diversion from axillary buds to the actively growing tip region of plants, which display a high sugar strength for photoassimilates (Kebrom, 2017). In one-node stem explant, where the actively growing tip is removed, our results reveal that the role of auxin could be extended to the regulation of the sink strength of axillary buds, and this effect is dependent on sugar availability. Vacuolar invertase (RhVI1), a marker of the growing capacity of buds in *Rosa hybrida* (Girault et al., 2010; Rabot et al., 2012) and in etiolated potato stems (Salam et al., 2021), is strongly repressed by auxin in dormant buds, and this repressive effect is partially compromised by increasing sugar availability from 10 to 100 mM. *RhVI1* constitutes a critical target of the combined effect of auxin and sugar at the transcriptional and protein (activity) levels (Figure 2), coinciding with its role as a hub for exogenous (light) and endogenous (sugar and GA) clues in *Rosa* buds (Rabot et al., 2014). Auxin exogenously fed to buds also results in the repression of *Rosa hybrida Sucrose transporter* (*RhSUC2*), a main plasma membrane-located sugar transporter that provides sugar to buds in the early stages of their outgrowth (Henry et al., 2011). Auxin-induced bud dormancy goes together with upregulated sugar limitation-related markers, including *ATAF1* and *trehalase* (Figure 3), *Asparagine synthetase 1* (*RhASN1;* Wang et al., 2021), and downregulation of two main primary pathways of the sugar metabolism (glycolysis/the TCA cycle and the OPPP; Wang et al., 2021). We might assume that besides its systemic role, auxin acts locally in the vicinity of buds in the stem by limiting their sink strength for sugar, leading to sugar depletion and bud dormancy whose extent may be compromised by sugar availability in the plant.

Besides the sugar metabolism, nitrogen availability is required for bud outgrowth in many species, including *Rosa* sp. (Le Moigne et al., 2018; Luo et al., 2020). The present study shows that the amino acid metabolism is impaired in dormant buds, with an elevated level of 17 transcripts encoding amino-acid-degrading enzymes and a low level of nine amino acids (Ala, Asp, Met, Glu, Gln, Pro, Lys, His, and Gly; Figure 4). Once again, rising sugar availability partially compromised the effect of auxin, indicating that the amino acid metabolism is a target of these two shoot branching regulators. More interestingly, six out of nine of these amino acids (Ala, Asp, Glu, Gln, Met, and His) were the same as those induced in response to plant decapitation in pea, reflecting the ability of buds to synthesize certain amino acids (Fichtner et al., 2017). In rose, several amino acids including Asn, Glu, Ser, Thr, Ile, and Pro accumulate in the xylem sap following decapitation, and both Asn and sugar are required for sustained growth of secondary axes (Le Moigne et al., 2018). The removal of the actively growing tip of a plant likely goes together with changes in the amino acid metabolism in buds and roots (Fichtner et al., 2017; Le Moigne et al., 2018). In our conditions, proline was the only amino acid whose accumulation was strongly enhanced (4.1 times) in response to sugar availability (from 10 mM to 100 mM sucrose) and plummeted in dormant buds (Figure 4). High levels of proline and alanine (Figure 4) can activate TOR kinase, which in turn represses proline consumption for cell respiration by mitochondria and stimulates its accumulation for protein synthesis (O’Leary et al., 2020). The isoleucine (Ile) and lysine (Lys) content was stimulated in dormant buds and inhibited in active ones (Figure 4), in line with their decreasing trend in bud-decapitated pea plants (Fichtner et al., 2017). Enhanced Lys metabolism negatively interacts with TCA cycle-associated metabolism during early germination (Angelovici et al., 2011). The carbon and nitrogen metabolisms are required for sulfur availability by providing OAS, the sole entry point of reduced sulfur in the form of sulfide into the plant metabolism (Hawkesford and De Kok, 2006; Jobe et al., 2019). Sulfur is an essential nutrient for all organisms and regulates plant growth *via* glucose-TOR signaling (Dong et al., 2017). In addition, the sulfur metabolism leads to the formation of glutathione (GSH), an important player in the redox status of plants (Ahmad et al., 2016; Olson, 2020). The coordination of the sulfur flux between GSH biosynthesis and protein translation determines growth *via* TOR regulation (Speiser et al., 2018). Dormant buds exhibited downregulated transcript levels of APS and APR, along with a low content of OAS (a sulfur precursor) and of the main sulfur-containing compounds including Cys, homoserine, and methionine, compared to active ones (Figure 5). Depletion of OAS inhibits TOR activity by downregulating the glucose metabolism, resulting in decreased protein translation and meristematic activity, and elevated autophagy (Dong et al., 2017). The global cell metabolism of buds, covering the sugar, amino acid, and sulfur metabolisms, is altogether more likely to be a potent target of the combined effect of auxin and sugar. This opens the way for future research aimed at identifying the network involved in this regulation.

We previously showed that the antagonistic effect of auxin and sugar availability mainly targeted SL signaling, and sugar did not antagonize auxin regarding CK synthesis in the stem (Bertheloot et al., 2020). Auxin triggers SL signaling to inhibit bud outgrowth, while sugar counterbalances this effect. Interestingly, similar trends were found for the auxin, ABA, and CK signaling pathways in buds (Figure 8), with a marked tendency for the ABA and CK signaling pathways (Figure 8). Dormant buds exhibited positive changes in auxin (YUCCA) transport and signaling, leading to elevated levels of SAUR, representing the key early auxin response genes (Ren and Gray, 2015; Stortenbeker and Bemer, 2019), in accordance with low auxin accumulation (Barbier et al., 2015). The ABA signaling pathway seems to be further at the core of this crosstalk because it involves all the components of ABA signaling including ABA perception (PYR/PYL), transduction (PP2C, SnRK2), and the expression of two ABA-dependent transcription factors (ABF2 and ABF4; Figure 8B). Dormant buds maintain a high ABA signaling pathway. ABA is a potent growth inhibitor (Yao and Finlayson, 2015) that was significantly compromised by elevated sugar availability (Figure 8B). Upregulation of *ABF2* and *ABF4* in dormant buds is in line with their role, along with ABF3, in the promotion of ABA-mediated chlorophyll degradation and leaf senescence in arabidopsis (Gao et al., 2016). This could prevent buds from acquiring a photosynthetic capacity following their outgrowth. ABA accumulation in dormant buds is triggered by BRC1 transcriptional activity. Along with three transcription factors *HB21*, *HB40*, and *HB53*, BRC1 upregulates the expression of NCED3, one of the main ABA biosynthesis enzymes, and thereby ABA signaling (González-Grandío et al., 2017). BRC1 is also under the control of sugar alone (Mason et al., 2014; Barbier et al., 2015; Wang et al., 2019) and of the combined effect of auxin and sugar (Wang et al., 2021). It would be very interesting to investigate whether auxin and sugar could regulate ABA signaling through BRC1-dependent and independent mechanisms. CK signaling in buds is also antagonistically affected by auxin and sugar: type-*B RR1* was upregulated by auxin and downregulated by sucrose, while type-*A RR3*, *5*, and *9* were downregulated by auxin and upregulated by sucrose (Figure 8C). Although this effect seems to be at odds with the promotive role of CK in shoot branching, it is perfectly consistent with previous data on arabidopsis. Indeed, the sextuple mutant lacking the whole clade of type-A Arabidopsis Response Regulators (*arr 3*,*4*,*5*,*6*,*7*,*15*) showed reduced shoot branching, while the *arr1* mutant exhibited high shoot branching, compared with the wild type (Müller et al., 2015; Waldie and Leyser, 2018). These phenotypes are correlated with the opposite effects of ARRs on stem auxin transport and the auxin export proteins PIN3, PIN4, and PIN7 (Müller et al., 2015; Waldie and Leyser, 2018), contributing to the connective auxin transport between bud and stem (van Rongen et al., 2019). All these findings pave the way for future investigations of the molecular mechanistic regulation of these hormone-signaling pathways in buds.

In plants, TOR kinase is known as the master regulator of growth that integrates diverse nutrient, energy, hormone, and stress inputs and transduces them into the regulation of ribosome biogenesis, cell cycle progression, leaf sink-to-source transition, cell growth, and autophagy (Ahn et al., 2015; Xiong and Sheen, 2015; Brunkard et al., 2020; McCready et al., 2020). TOR kinase is a player in the meristematic activity of roots and apical buds (Li et al., 2017; Wu et al., 2019); our results reveal its role in the control of bud outgrowth. Axillary bud treatment with AZD8055, a potent inhibitor of TOR kinase (Schepetilnikov et al., 2017; Zhuo et al., 2020), resulted in a reduction of their ability to grow out in a concentration-dependent manner (Figure 9). Furthermore, two downstream TOR-kinase processes—ribosome biosynthesis and DNA replication—were strongly affected by the antagonistic effect of sucrose and auxin (Figure 7). The overwhelming majority of the transcript levels of the genes related to ribosome biosynthesis (27 genes) and DNA replication (19 genes) was highly repressed in auxin-related dormant buds, and this effect was significantly compromised by sugar availability (Figure 7). This is further in accordance with the fact that these two processes are among the most energy-consuming ones, and dormant buds are highly energy-limited sink organs (Wang et al., 2021). TOR-kinase-dependent regulation of ribosome biosynthesis and DNA replication could be triggered by the phosphorylation of its downstream targets S6 kinase and eIF4E binding protein 1 (E-BP1; Sablowski and Carnier Dornelas, 2014; Xiong and Sheen, 2014), allowing TOR kinase to coordinate ribosome biogenesis with nucleotide availability to maintain metabolic homeostasis and support plant growth (Busche et al., 2021). Recently, Scarpin et al. (2020) showed that TOR controls ribosome biogenesis at several steps, including through phosphorylation of LARP1, which is a conserved TOR substrate. Much evidence indicates that auxin directly stimulates TOR kinase in root and shoot to promote their growth (Bögre et al., 2013; Xiong et al., 2013; Li et al., 2017; Schepetilnikov and Ryabova, 2017). Our findings assume that the located-stem auxin, that cannot enter bud to exert its inhibitory effect, indirectly represses TOR kinase activity *via* the regulation of branching-related hormones (CK or SL) and/or of bud sugar starvation. Additional studies are required to decipher the underlining mechanism behind this regulation.

TOR kinase is more likely to act negatively on and upstream of BRC1, as evidenced by the upregulation of BRC1 and *RhHB40*, a marker of its transcriptional activity (González-Grandío et al., 2017; Wang et al., 2021), in response to AZD8055. This effect was negatively dependent on sugar availability (Figure 9) and took place at the promoter level of *RhBRC1* (Figure 9). Along with the fact that glycolysis/TCA cycle- and OPPP-dependent signaling pathways cooperatively regulate *RhBRC1* expression at its promoter level (Wang et al., 2021), these findings clearly indicate that the promoter region of *RhBRC1* is the converging site of several endogenous signals and could be targeted to disentangle the molecular regulatory mechanisms that drive BRC1 regulation.

The antagonistic effect of two main systemic regulators of shoot branching—auxin and sucrose availability—operates through a complex network of processes based on the cell metabolism and signaling pathways. Part of these processes act upstream of TOR kinase, while others are directly related to its activity, consistent with the activity of TOR kinase required for bud outgrowth. These findings open up new avenues for a better understanding of the way(s); these two main endogenous regulators are integrated in buds and drive their ability to turn into a new branch, an important trait for agronomy, and horticulture.

## Data Availability Statement

The original contributions presented in the study are publicly available. This data can be found at: National Center for Biotechnology Information (NCBI) BioProject database under accession number PRJNA508209.

## Author Contributions

SS supervised this work. MW and SS conceived all experiments. MW, LO, and M-DP carried out different experiments. AL-A carried out RNA sequencing. GC performed the metabolomics approach. MW drew the figures. LO, JL, and LH contributed to the improvement and writing of the article. All authors contributed to the article and approved the submitted version.

## Funding

This research was funded by the China Scholarships Council (no. 201506320203), Well-Breed Engineering of Shandong province (2021LZGC022), Talent Introduction Special Funds of Qingdao Agricultural University (663/1120070), and the Agence Nationale de la Recherche (ANR) project Labcom, called ESTIM (Evaluation de STIMulateurs de vitalité des plantes).

## Conflict of Interest

The authors declare that the research was conducted in the absence of any commercial or financial relationships that could be construed as a potential conflict of interest.

## Publisher’s Note

All claims expressed in this article are solely those of the authors and do not necessarily represent those of their affiliated organizations, or those of the publisher, the editors and the reviewers. Any product that may be evaluated in this article, or claim that may be made by its manufacturer, is not guaranteed or endorsed by the publisher.
