# Supplementary material for: Antagonistic Effect of Sucrose Availability and Auxin on Rosa Axillary Bud Metabolism and Signaling, Based on the Transcriptomics and Metabolomics Analysis
Source: Front Plant Sci. 2022 Mar 17;13:830840. doi: 10.3389/fpls.2022.830840 (PMC8982072; doi:10.3389/fpls.2022.830840)
Supplement: Supplementary file 1 [file Table_1.pdf]

Table S1. Primers used for quantitative real time PCR (qPCR)

Gene	Primer sequence	
RhASN1	Forward	5'-CTATTCGAGCCAGCACCCC-3'
	Reverse	5'-TCTCATCAGAGCCCTCACCAG-3'
RhSTP1	Forward	5'-TAAAGGGCGGTTGGGGATG-3'
	Reverse	5'-GGTCTTGGCTTTCTCGTGCTG-3'
RhPFK	Forward	5'-GTGGTTATGATTTGCTTGGACG-3'
	Reverse	5'-TCAGTGTTTGATGTCACCCCTC-3'
RhPK	Forward	5'-TTCACCCAACCCCAACCA-3'
	Reverse	5'-CAGCGGATACTTTCCATTAGCA-3'
RhMDH	Forward	5'-GGACTCCTCCCGCTTTCG-3'
	Reverse	5'-GTGCCACTGAGCTATCACCATG-3'
RhPGD	Forward	5'-AGAGTGCTGCTCGTATGATTGC-3'
	Reverse	5'-TTGATGCCTTTGCTGTTGTGA-3'
RhG6PD	Forward	5'-GGCTTTACGGTTGGGACATT-3'
	Reverse	5'-TCTGAAAACCCCGACTGCTC-3'
RhHB40	Forward	5'-TTCGGCAACGAGCATAAACTG-3'
	Reverse	5'-TTCTGAAACCAAACAGCCACTT-3'
RhOGDH	Forward	5'-GATGGCTGGAGGTTCATTTACA-3'
	Reverse	5'-TGCCGACTGAGGAGGGTTG-3'
RhHXK1	Forward	5'-GTTGGGACCAAACTCAAGGA-3'
	Reverse	5'-TGGCAACTACGTCGCATAAC-3'
RhBRC1	Forward	5'-TGCATTGTTTAACCCTCTTGCA-3'
	Reverse	5'-GTTCTTTCTCTTGTCTCGCTCTT-3'

RhHXK1, hexokinase in *Rosa hybrida*; *RhPFK*, 6-phosphofructokinase in *Rosa hybrida*; *RhPK*, pyruvate kinase in *Rosa hybrida*; *RhOGDH*, 2-oxoglutarate dehydrogenase in *Rosa hybrida*; *RhMDH*, malate dehydrogenase in *Rosa hybrida*; *RhG6PD*, glucose-6-phosphate 1-dehydrogenase in *Rosa hybrida*; *RhPGD*, 6-phosphogluconate dehydrogenase in *Rosa hybrida*; *RhASN1*: *Rosa hybrida* asparagine synthetase 1; *RhSTP1*: *Rosa hybrida* sugar transporter protein 1, *RhBRC1*: *Rosa hybrida* BRANCHED1, *RhHB40*: a positive marker of the transcriptional activity of *BRC1*.
