# Supplementary material for: Antagonistic Effect of Sucrose Availability and Auxin on Rosa Axillary Bud Metabolism and Signaling, Based on the Transcriptomics and Metabolomics Analysis
Source: Front Plant Sci. 2022 Mar 17;13:830840. doi: 10.3389/fpls.2022.830840 (PMC8982072; doi:10.3389/fpls.2022.830840)
Supplement: Supplementary file 2 [file Table_2.pdf]

Table S2. Primers used to subclone 1973pb-*RhBRC1* promoter

Primer name	Primer sequence
ProRhBRC1-1F	5’-CACCTGTGAGCTAGTTGAGAAAACAATTG-3'
ProRhBRC1-R	5’-TGTGATGTATATAGCTAATATCTGGTTG-3'
