# Supplementary figures and images for: Antagonistic Effect of Sucrose Availability and Auxin on Rosa Axillary Bud Metabolism and Signaling, Based on the Transcriptomics and Metabolomics Analysis
Source: Front Plant Sci. 2022 Mar 17;13:830840. doi: 10.3389/fpls.2022.830840 (PMC8982072; doi:10.3389/fpls.2022.830840)

A

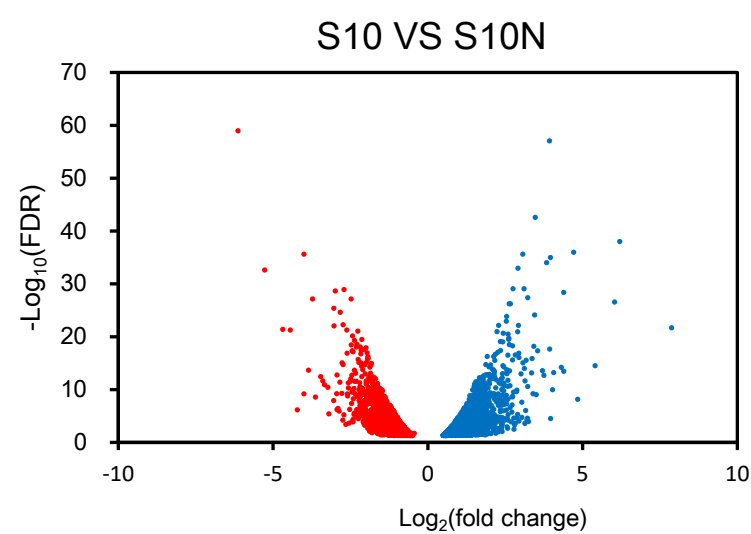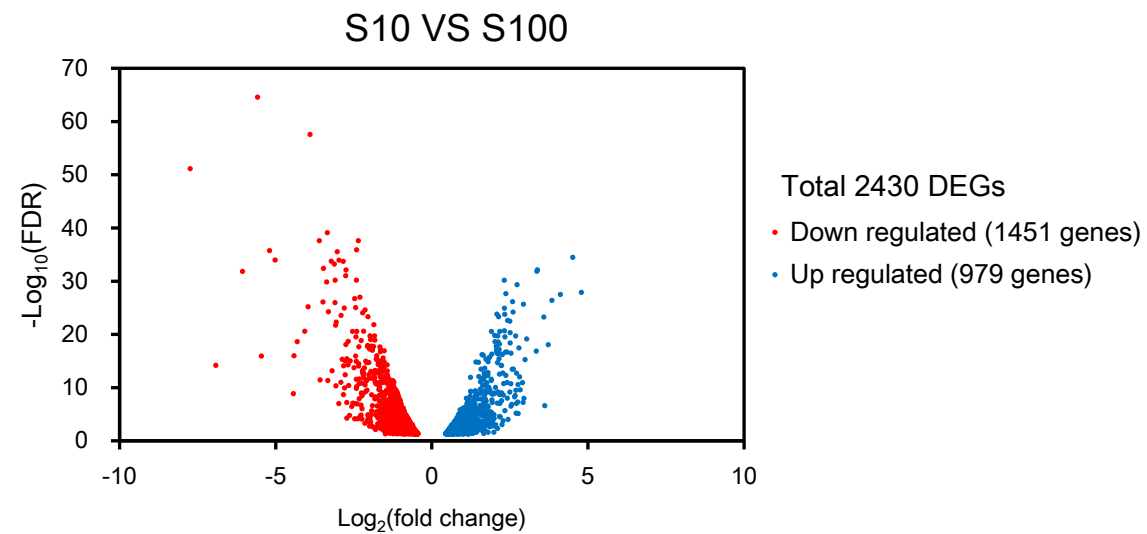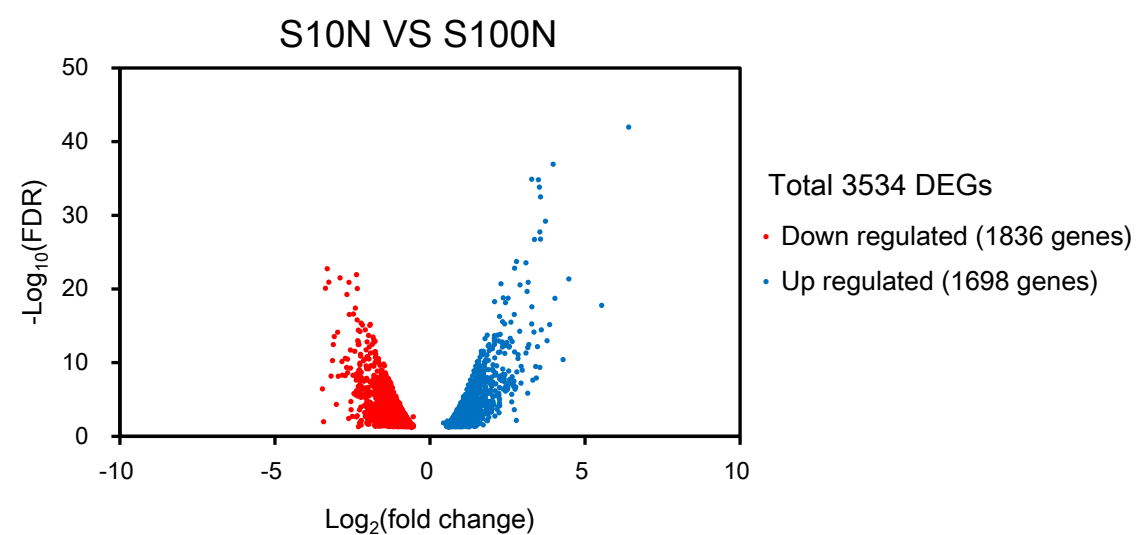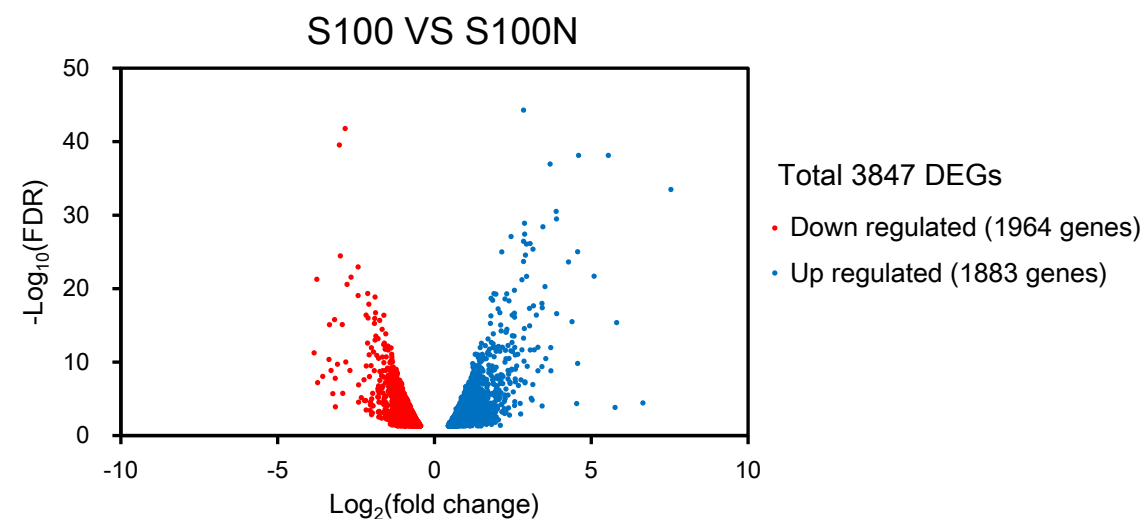

Supplement: Supplementary file 3 [file Image_1.pdf]

B

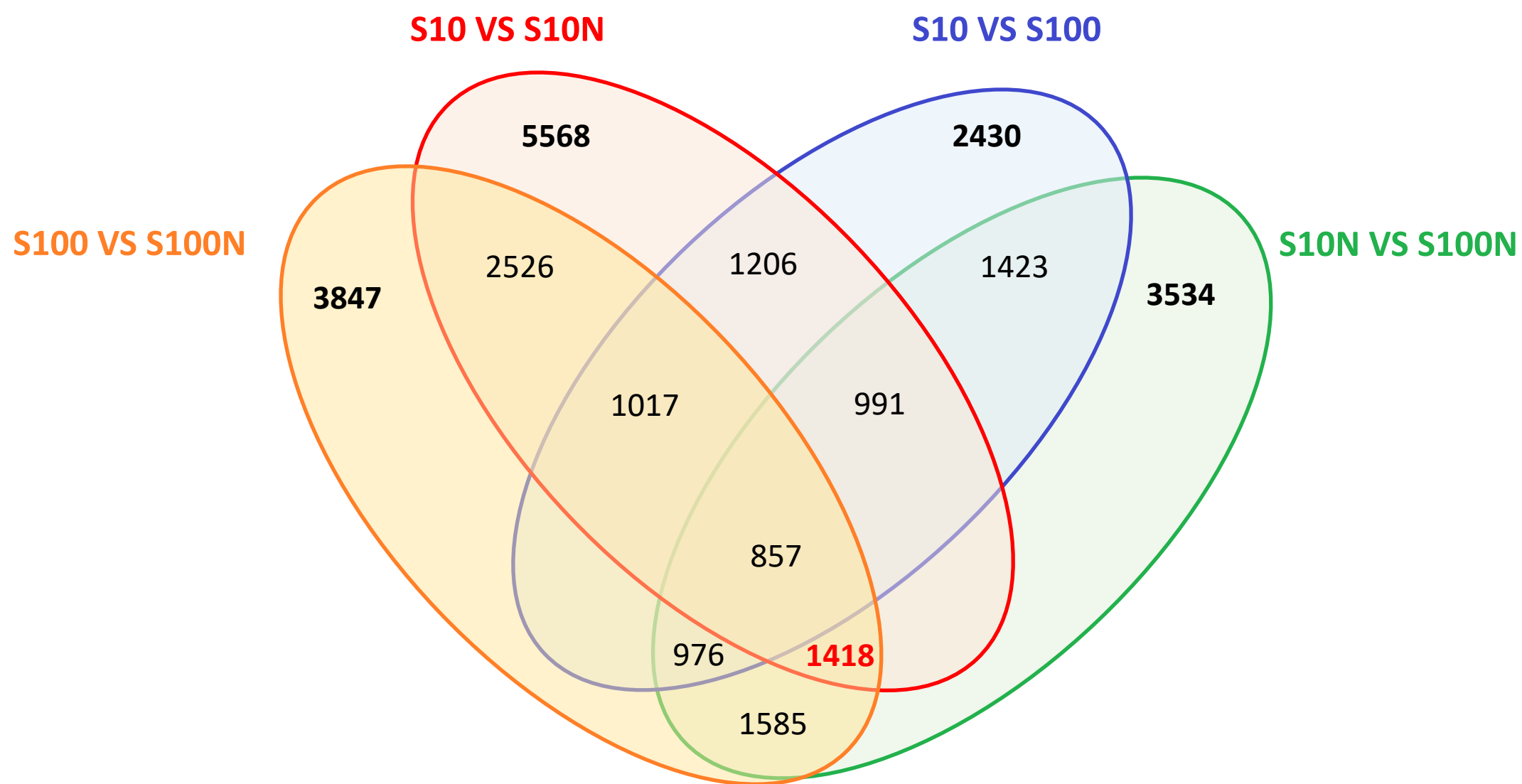

Supplement: Supplementary file 4 [file Image_2.pdf]

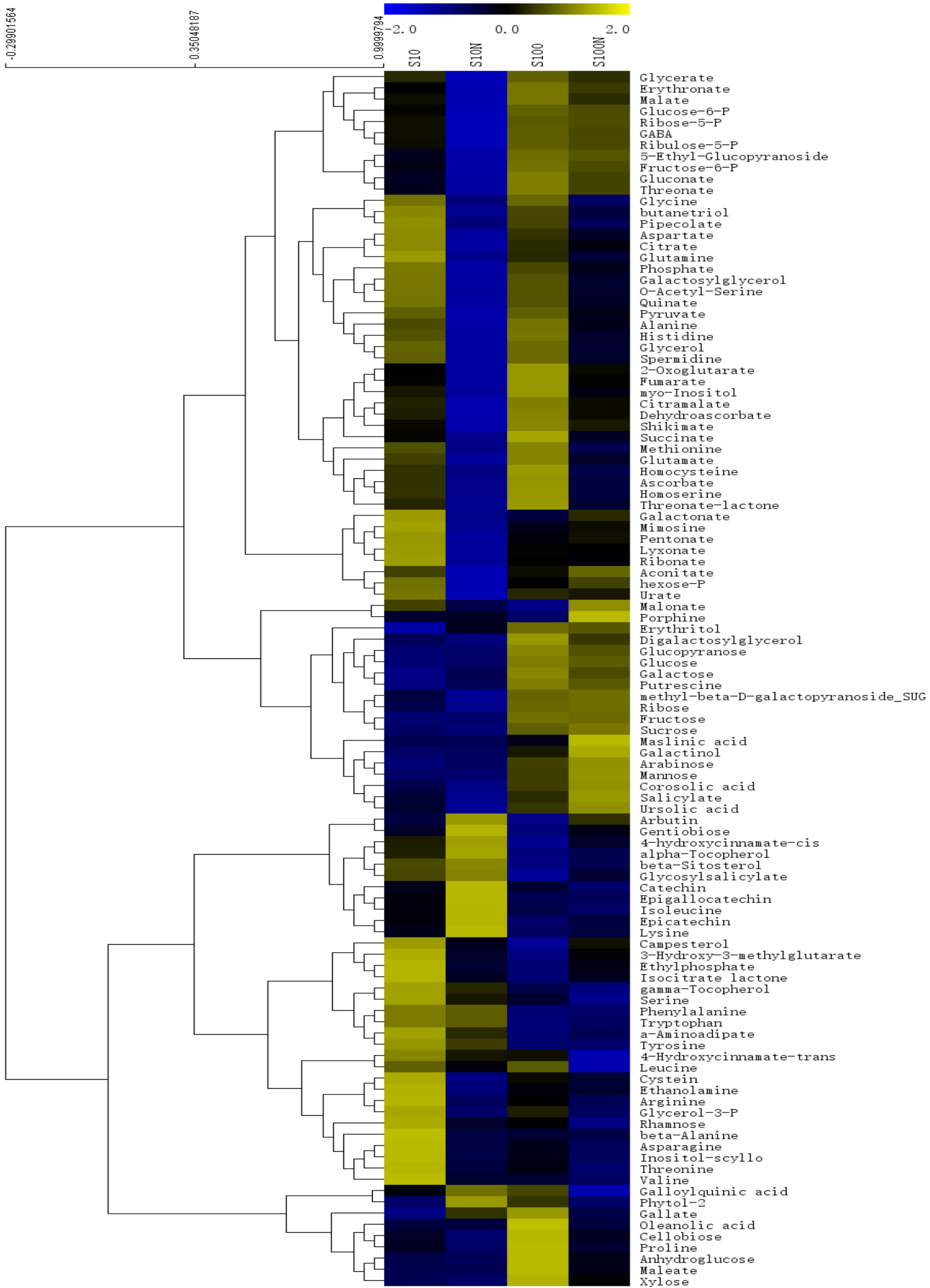

Supplement: Supplementary file 5 [file Image_3.pdf]

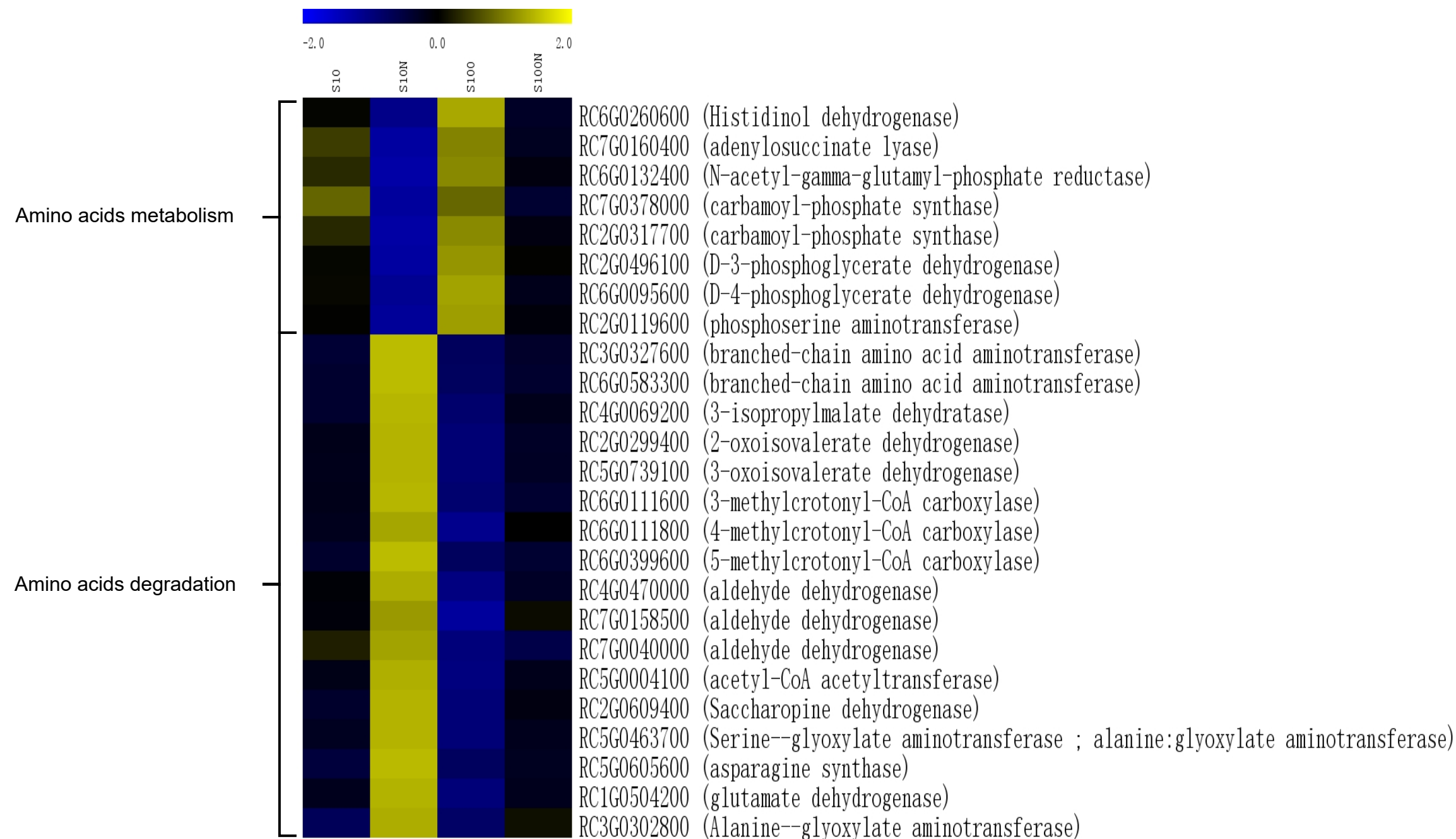

Supplement: Supplementary file 6 [file Image_4.pdf]

Ribose-5-P

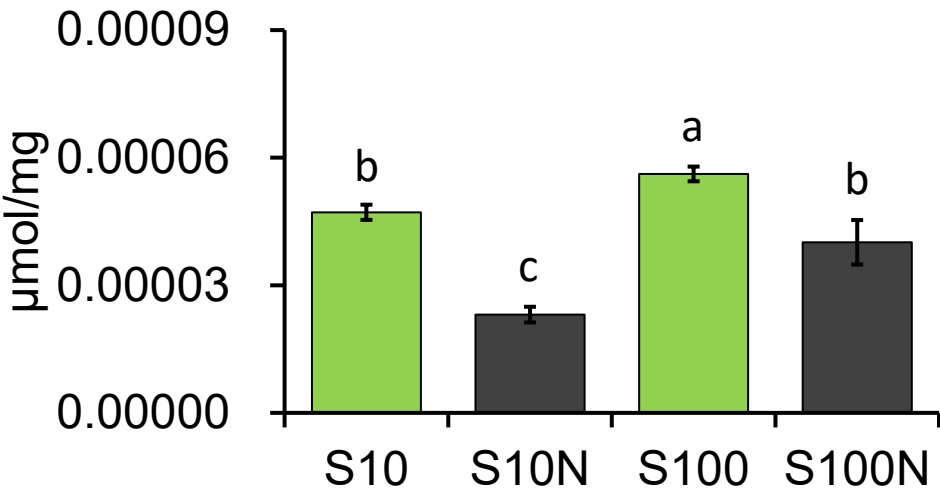

Supplement: Supplementary file 7 [file Image_5.pdf]
